# Development of a nanoparticle-based immunotherapy targeting CD137 for nasopharyngeal carcinoma treatment

**DOI:** 10.7150/thno.116136

**Published:** 2026-01-01

**Authors:** Kang Yi Lee, Heng Sun, Yu Mei, Emily Nickles, Clemence Wei Xia Lai, Sashigala Ponnalagu, Mukul Prasad, Haiyan Liu, Herbert Schwarz

**Affiliations:** 1 NUS Immunology Programme, Life Sciences Institute, Department of Microbiology and Immunology, National University of Singapore, 117545 Singapore.; 2 NUSMED Immunology Translational Research Programme, National University of Singapore, 117456 Singapore.; 3 Department of Diagnostic Radiology, Yong Loo Lin School of Medicine, National University of Singapore, 119074 Singapore.; 4 Department of Physiology, Yong Loo Lin School of Medicine, National University of Singapore, 117593 Singapore.; 5 Department of Haematology-Oncology, National University Cancer Institute, Singapore (NCIS), National University Hospital, Singapore, Singapore.; 6 Yong Loo Lin School of Medicine and Cancer Science Institute (CSI), National University of Singapore (NUS), Singapore, Singapore.

**Keywords:** CD137, T cell-based immunotherapy, cancer immunotherapy, NPC, mesoporous silica nanoparticles

## Abstract

CD137 is a powerful T cell costimulatory molecule, and CD137 agonists are being evaluated for human cancer immunotherapy. Urelumab and utomilumab, are two agonistic anti-CD137 antibodies that are most advanced in clinical trials but suffer from liver toxicity and low potency, respectively. Here we describe the development of a new type and formulation of a CD137 agonist that combines high potency and a strong safety profile.

**Methods:** The extracellular domain of recombinant human CD137 ligand (rhCD137L) was conjugated onto mesoporous silica nanoparticles (MSNs) of approximately 50 nm in diameter, and the ratio of rhCD137L to MSNs was optimized based on their ability to costimulate the cytotoxic activity of T cells. As nasopharyngeal carcinoma (NPC) cells often express CD137, the *in vitro* effect of rhCD137L-MSNs on T cell-mediated tumor cytotoxicity was evaluated using the NPC cell lines C666 and HK-1, each tested as CD137-expressing and -deficient variants. Results were compared with those obtained using MSNs conjugated with urelumab (ure-MSNs) or unconjugated urelumab. The biodistribution, therapeutic efficacy and toxicity of rhCD137L-MSNs were subsequently assessed in humanized mouse NPC models.

**Results:** rhCD137L-MSNs were of higher potency than ure-MSNs or unconjugated urelumab in inducing *in vitro* T cell killing of the NPC cell lines C666 and HK-1, of both CD137-expressing and -deficient phenotypes. C666-CD137 and HK1-CD137 cells were eliminated more efficiently than the CD137-deficient cells. *In vivo*, in humanized mouse NPC models, both rhCD137L-MSNs and ure-MSNs inhibited tumor growth, with rhCD137L-MSNs being slightly more potent. This was reflected in an increase in T cell activation markers and an increased infiltration of effector memory CD8^+^ T cells into the tumor. In contrast to ure-MSNs, rhCD137-MSN treatment did not induce liver damage, thereby demonstrating a more favorable safety profile than ure-MSNs.

**Conclusions:** This study identifies a formulation of rhCD137L on MSNs that combines high potency with excellent safety.

## Introduction

Immunotherapy has transformed cancer treatment approaches by harnessing the power of the immune system to target and eliminate malignant cells. Among the key modulators of immune activation, CD137 (TNFRSF9, 4-1BB), a member of the tumor necrosis factor receptor superfamily, has garnered significant interest since its discovery in the late 1980s 1, 2. CD137 is expressed on T cells and natural killer (NK) cells upon activation 3, 4. Under physiological conditions, the engagement of CD137 with its natural ligand (CD137L) on antigen presenting cells (APCs) activates downstream signaling cascades, including the NF-κB, ERK, JNK and PI3K/Akt pathways 5-7.

Activation of CD137 signaling enhances T cell survival, proliferation, differentiation to effector memory cells and production of Th1-associated cytokines, such as IFNγ and TNF 8-13. In addition, CD137 agonism synergistically enhances PD-1 blockade-mediated reinvigoration of anti-tumor CD8^+^ T cells 14. However, initial clinical trials faced challenges in striking a balance between the efficacy and safety of the CD137 agonists. The CD137-specific monoclonal antibody urelumab is potent but can cause severe grade 3-4 hepatotoxicity, whereas utomilumab is safe but has low efficacy as a monotherapy even at doses up to 10 mg/kg 15, 16. As a result, their clinical development was discontinued. Hence, many attempts, including tumor-targeted bispecific antibodies, are currently underway to potently costimulate effector T cells while mitigating liver damage.

While effector T cell costimulation has been the focus of CD137-based therapy, we demonstrated that some cancer cells ectopically express CD137 as that gives them survival advantages. In Hodgkin lymphoma (HL), Epstein-Barr virus (EBV)-induced CD137 expression on Hodgkin and Reed-Sternberg (HRS) cells enhances TNF, IL-6 and IL-13 production, which are further heightened upon CD137 stimulation 17, 18. Similarly, CD137 is ectopically expressed on nasopharyngeal carcinoma (NPC) and rhabdomyosarcoma (RMS) cells, and CD137 signaling induces their secretion of IL-6 and IL-8 which promotes metastasis and indicates a poor prognosis 19, 20. In addition, CD137 on these cancer cells usurps a negative feedback mechanism of CD137 which is used under physiological conditions to prevent T cell over-activation and autoimmune damage. During this process, CD137 is trogocytically transferred from cancer cells to CD137L on APCs, resulting in the endocytosis of the receptor-ligand complex and thus impairing the CD137L-mediated costimulatory activity of APCs 19-21. In addition, CD137 is expressed on NK/T cell lymphoma (NKTCL), chronic lymphocytic leukemia (CLL), and follicular dendritic cell (FDC) lymphoma 22-24. Despite these findings, the therapeutic potential of CD137 on cancer cells has so far not been addressed.

Mesoporous silica nanoparticles (MSNs) have recently emerged as promising carriers for immunomodulatory proteins and vaccines in cancer treatment, due to their high biocompatibility, tunable structural properties and ease of surface functionalization 25, 26. MSNs loaded with tumor-associated antigens (TAAs) promote antigen presentation by dendritic cells (DCs) 27. Moreover, the use of MSNs to co-deliver anti-PD-L1 antibody and a polo-like kinase 1 (PLK1) inhibitor was found to enhance lung cancer susceptibility to PD-L1 blockade 28. Liu and colleagues also demonstrated that MSNs encapsulating anti-PD-L1 antibody, and a Ying-yang-1 inhibitor could target multiple pathological aspects of hepatocellular carcinoma (HCC) 29.

In this study, we conjugated recombinant human CD137L (rhCD137L) to MSNs via the formation of peptide bonds, thus rendering it multimeric. Our data show that rhCD137L-MSNs induce robust T cell costimulation, driving stronger tumor cytotoxicity than unconjugated urelumab or MSNs conjugated with urelumab (ure-MSNs) or utomilumab (uto-MSNs), the clinically most advanced anti-CD137 antibodies. Also, rhCD137L-MSNs in contrast to ure-MSNs show reduced liver toxicity. More importantly, the *in vitro* effect of rhCD137L-MSNs is particularly pronounced when tumor cells express CD137, highlighting that dual targeting of CD137 on both tumor cells and T cells synergistically maximizes the T cell-mediated anti-tumor immune response. In summary, we describe a CD137 agonist with high potency and lack of toxicity as an alternative to conventional CD137-specific antibodies. This agonist gains even greater efficacy when cancer cells express CD137 that facilitates their pathophysiology and escape from immune surveillance.

## Materials and Methods

### Mice

NOD.Cg-*Prkdc^scid^/*JInv (NOD scid - Jax) mice (aged 6-7 weeks) were purchased from InVivos (Singapore) for the *in vivo* evaluation of the therapeutic effects and potential adverse effects of rhCD137L-MSNs. Mice were housed in a specific pathogen-free facility and were handled in compliance with the approved animal protocol by the Institutional Animal Care and Use Committee (IACUC) of National University of Singapore (NUS), Singapore.

### Cell lines

Human NPC cell lines C666 and HK1 were gifts from Dr. Paul MacAry and Dr. Zhang Yongliang (NUS, Singapore), respectively. C666 and HK1 cells stably were transduced with either full length CD137-encoding (C666-CD137 and HK1-CD137) or empty pLenti6/V5-D-TOPO vector (C666 and HK1). All cell lines were cultured in RPMI1640 (Sigma Aldrich, MO, USA) supplemented with 10% heat inactivated fetal bovine serum (FBS) (Biowest, MO, USA), 10 mM HEPES (Hyclone, IL, USA), 1 mM sodium pyruvate (Hyclone), MEM Non-Essential Amino Acids (Life Technologies, MA, USA), Penicillin-streptomycin (Hyclone), and beta-mercaptoethanol (Sigma Aldrich). The media is referred to as R10. These cell lines were passaged every two days and cultivated in a humidified chamber with 5% CO_2_ at 37 °C.

### Isolation of human PBMC and T cells

All human blood samples were obtained from healthy donors at Health Sciences Authority Singapore with approval from the Institutional Review Board, Singapore (IRB number: 2021-856) in accordance with the guidelines of the Health Sciences Authority of Singapore. PBMC were isolated by layering the blood samples on Ficoll-Pague media solution (GE Healthcare, IL, USA), followed by density gradient centrifugation.

Removal of residual erythrocytes was then done using ACK lysis buffer (Life Technologies). For T cell isolation, PBMC were resuspended in phosphate buffered saline (PBS) supplemented with 2% FBS and 1 mM EDTA at a cell density of 5 x 10^7^ cells/mL. The isolation procedure was done using EasySep^TM^ human CD3 T cell isolation kit (STEMCELL technologies, Vancouver, Canada) as per manufacturer's protocol. Isolated human T cells were resuspended in R10 for further use.

### Antibodies and proteins

The agonistic anti-CD137 antibodies urelumab and utomilumab, as well as human IgG4 (huIgG4) isotype control were purchased from iChorBio (Oxfordshire, UK). Bovine serum albumin (BSA) was purchased from Biowest.

### Construction of rhCD137L plasmid

A DNA sequence encoding for TwinStrep-tag, TEV sequence and extracellular human CD137L (position 50 - 254) was cloned into pTwist Amp high copy vector by Twist bioscience. TwinStrep-tag (SAWSHPQFEKGGGSGGGSGGSAWSHPQFEK) and TEV sequence (ENLYFQG) were added to the N-terminus of the extracellular human CD137L sequence. The DNA sequence of extracellular human CD137L was codon optimized. The insert (TwinStrep-TEV-extCD137L) was isolated by restriction enzyme digestion, purified and ligated into the pYD7 vector via the same restriction enzymes (XbaI and HindIII) to form a recombinant vector designated pYD7-TwinStrep-TEV-extCD137L. Transformation and colony PCR were performed to ensure successful insertion into the pYD7 vector before using the plasmid for protein expression.

### rhCD137L protein expression

Transient transfection was performed using HEK 293-6E cells (NRCC), which were cultured in FreeStyle F17 (Life Technologies). In short, pYD7-TwinStrep-TEV-extCD137L plasmid DNA was transfected into HEK 293-6E cells using PEI at a PEI:DNA ratio of 3:1. Supplement 20% Tryptone-N1 was added 24 h post transfection to stop the transfection process. On day 5 post transfection, culture supernatants were harvested by centrifugation, filtered through a 0.22 mm filter unit (Merck, Darmstadt, Germany) and loaded onto a Strep-Tactin XT column (IBA Lifesciences, Gӧttingen, Germany) for purification of rhCD137L protein. The purified protein was then subjected to buffer exchange using Vivaspin centrifugal concentrator (Cytiva, MA, USA) and concentrated in PBS, pH 7.2. Protein concentration was quantified using Nanodrop at 280 nm by entering the corresponding protein size and extinction coefficients.

### Synthesis of MSNs and conjugation of rhCD137L to MSNs

43 mL of deionized water containing 1 g of cetyltrimethylammonium chloride (CTAC) (Sinopharm Co., Shanghai, China) and 0.12 g of triethanolamine (TEA) (Aladdin Co., Shanghai, China) were heated to 95 °C with stirring for 1 h. Subsequently, 3 mL tetraethyl orthosilicate (TEOS) (Aladdin Co.) was added dropwise to the reaction solution, and the mixture was maintained at 95 °C for 1 h. The resulting MSNs were washed with 100% ethanol and were incubated with hydrochloric acid (HCl)-containing methanol at 60 °C overnight to remove residual CTAC. (3'-aminopropyl)trimethoxysilane (APTES) (Aladdin Co.) was then added to decorate the MSN surfaces with amine groups. MSNs were washed with nuclease-free HyPure water (Hyclone), and were mixed with rhCD137L, anti-CD137 antibodies (urelumab, utomilumab) and huIgG4 isotype control at a mass ratio of 3:10, following incubation for 48 h at 4 °C with continuously stirring. Afterwards, the protein-MSNs were pelleted via centrifugation. The supernatants were harvested, and the protein-MSN pellets were resuspended to desired concentrations in PBS.

### Conjugation of rhCD137L protein to Strep-Tactin® XT 4Flow® high-capacity resin beads

rhCD137L protein at a fixed amount of 312.5 nM was mixed with Strep-Tactin® XT 4Flow® (ST) high-capacity resin beads (IBA Lifesciences) to a final concentration of 0.016, 0.16, 0.64, 1.28, 5.12 or 20.48 mM for the resin beads. PBS was used as a diluent. The protein-resin bead mixtures were incubated on a rotator for 2 h at room temperature to generate rhCD137L-ST conjugates.

### Real-time cytotoxicity assay using xCELLigence

The xCELLigence RTCA DP and SP (ACEA Biosciences, CA, USA) were used to monitor human T cell-mediated cytotoxicity against tumor cells. In brief, human NPC cell lines (C666, C666-CD137, HK1 or HK1-CD137) were seeded at a cell density of 1.5x10^4^ cells in 100 μL R10 per well in duplicates. The cell growth was monitored every 15 min. Meanwhile, freshly isolated human T cells were suboptimally activated with 1 μg/mL plate-bound anti-human CD3 antibody (clone: OKT3, Biolegend, CA, USA) and 2.5 μg/mL soluble anti-human CD28 antibody (clone: 37.51, eBiosciences, CA, USA) in the presence of 100 IU/mL human IL-2 (PeproTech, NJ, USA) for 18-20 h.

At 24 h post NPC cell seeding, the suboptimally activated T cells were added at a cell density of 9x10^4^ cells in 50 μL R10 supplemented with 400 IU/mL human IL-2 (PeproTech) for each corresponding well. The final E:T ratio was 6:1. For experiments using ST beads, rhCD137L-ST, unconjugated anti-CD137 antibodies (urelumab, utomilumab), huIgG4 isotype control antibody or rhCD137L were added to a final concentration of 70 nM. For experiments using MSNs, rhCD137L-MSNs, ure-MSNs, uto-MSNs, huIgG4-MSNs or naked MSNs were added at an equal molar concentration of receptor binding sites (197.5 nM). Control wells were established to include the following: target cells alone, target cell with treatment, effector cells alone, effector cells with treatment, and full lysis controls (0.25% Triton X). The data were expressed as percentage of cytolysis.

### T cell proliferation assay

Freshly isolated human T cells were labelled using CellTrace^TM^ CFSE Cell Proliferation Kit (Invitrogen, MA, USA), according to manufacturer's protocol. The cells were then seeded at a density of 5x10^5^ cells/mL per well into 96 well round-bottom plates, and stimulated with Dynabeads^TM^ Human T-Activator CD3/CD28 (Gibco, MA, USA) at a cell:bead ratio of 2:1 in the presence of 400 IU/mL human IL-2 (PeproTech). 24 h post stimulation, the cells were treated with 5 µg/mL of rhCD137L-MSNs or BSA-MSNs, and their proliferation was assessed three days later using flow cytometry.

### NPC cell line proliferation/viability assay

A total of 2x10^3^ C666 and C666-CD137 cells, and 3x10^3^ HK1 and HK1-CD137 cells were seeded per well into 96 well flat-bottom plates. The proliferation and viability of the NPC cells were determined using a Cell Counting Kit-8 assay (CCK8; Dojindo, Kumamoto, Japan) every 24 h for 3 consecutive days. To assess the effect of rhCD137L-MSNs on NPC cells, the cells were seeded for 24 h and subsequently treated with rhCD137L- or BSA-MSNs at 1, 5 or 10 μg/mL, before CCK8 assay. Briefly, 10 μL CCK8 solution was added to 100 μL of culture media, followed by one hour of incubation at 37 °C. Absorbance was then measured at 450 nm. Six replicates of each treatment were used, and experiments were performed in duplicates.

### Multiplex immunohistochemistry

Multiplex immunohistochemistry was performed on an NPC tissue microarray (NPC1506; US Biomax, MD, USA) according to manufacturer's protocol for opal multiplex immunofluorescent system (Perkin Elmer, Waltham, USA) and for PanPath (PanPath B.V., Amsterdam, The Netherlands). The tissues were probed with EBER oligo probe (PanPath B.V.) followed by HRP-SA (Perkin Elmer). Primary antibodies anti-CD137 (clone BBK-2; Thermo Scientific, MA, USA), or rabbit anti-CD3 (Dako, Glostrup, Denmark) were used followed by anti-mouse or anti-rabbit HRP polymers, respectively (GBI Labs, WA, USA). The tissues were developed with 4-color Opal IHC kit (Perkin Elmer) and nuclei staining was performed with DAPI. Tissue sections were imaged on Vectra Imaging system, and analyzed with inForm (Perkin Elmer).

Quantitative image analysis was performed using Qupath v0.5.1. For cell segmentation, pretrained StarDist models with default prediction parameters were used. Nuclear signals were derived from the summation intensities of DAPI and EBER channels, and were capped at the 99th percentile before segmentation. After segmentation, cells outside the 5th-95th percentile size range were excluded from downstream analysis. For cell phenotyping, a deep learning cell phenotyping algorithm (MAPs) as described in 30 was used, generating a trained phenotyping model which yielded a precision score and recall metric of >80% on ground truth data, and thus applied to the TMA. Tumor cores were assigned to categories based on the frequency of EBER^+^ or CD137^+^ EBER^+^ cells: low (<1%), intermediate (1-5%), and high (>5%).

### Western blot

To preserve the oligomerization status of rhCD137L on ST beads, 5 mM bis(sulfosuccinimidyl) suberate (BS3; Thermo Scientific) was mixed with 1 μg of rhCD137L-ST to crosslink the protein prior to Western blotting, followed by a 30 min incubation at room temperature. The crosslinking reaction was stopped with the addition of 1 M Tris-HCl (pH 8) to a final concentration of 50 mM, followed by a 15 min incubation at room temperature.

Protein concentrations were determined using a Bradford Protein Assay kit (BioRad). Protein samples were separated by sodium dodecylsulfate (SDS)-polyacrylamide gel electrophoresis (PAGE) and transferred to polyvinyldifluoride (PVDF) membranes according to standard protocols. The membranes were then blotted with an anti-CD137L primary antibody (clone: 5F4; Thermo Scientific) in 5% skim milk diluted with Tris-buffered saline (TBS) containing 0.1% Tween. Afterwards, the membranes were probed with Pierce^TM^ horseradish peroxidase (HRP)-conjugated goat anti-mouse secondary antibody (Thermo Scientific) and incubated with chemiluminescent substrates for imaging using Chemidoc^TM^ MP imaging system (Bio-Rad).

### *In vivo* studies

#### Humanized mouse NPC models

*In vivo* biodistribution and therapeutic studies were performed using humanized mouse NPC models. Humanized NOD-scid (JInv) mice were established by engrafting with 10^7^ freshly thawed PBMC via tail vein injection. After one week, mice were subcutaneously injected with 10^6^ NPC cells of wildtype (C666 or HK1) and CD137-overexpresssing (C666-CD137 or HK1-CD137) phenotype into their left and right flanks, respectively. Tumors were measured (length x width, mm^2^) every other day, and were allowed to grow to a size of 100 mm^2^ before mice were treated.

#### *In vivo* and *ex vivo* rhCD137L-MSN biodistribution study

To determine whether rhCD137L-MSNs can infiltrate NPC, NPC-bearing humanized mice were administrated with a single intravenous dose of 10 mg/kg of Cy5.5-labelled rhCD137L-MSNs or naked MSNs. 48 h post-injection, the *in vivo* biodistribution of rhCD137L-MSNs or naked-MSNs was visualized using the *In Vivo* Imaging System (IVIS; Revvity, MA, USA). Subsequently, tumors and major organs, including liver, kidneys, lungs and spleen, were also excised for ex vivo fluorescence imaging using IVIS.

#### rhCD137L-MSN therapeutic study

NPC-bearing humanized mice were randomly divided into four groups: PBS, huIgG4-MSNs, ure-MSNs and rhCD137L-MSNs. The nanoparticles were administrated for a total of three doses given at three-day intervals. Endpoint criteria for euthanasia were defined by a body-weight reduction of 20%. Three days after the last treatment, mice were sacrificed.

#### Hepatotoxicity measurement

To study the hepatotoxic effect, fresh liver tissues were homogenized using Omni bead ruptor (Omni, SF, USA). Supernatants of the tissue homogenates were tested for their total protein concentration using Pierce^TM^ BCA protein assay kit (Thermo Scientific). Alanine aminotransferase (ALT) and aspartate aminotransferase (AST) detection were performed using ALT/GPT activity kit (Invitrogen) and AST/GOT activity kit (Invitrogen), respectively, as per manufacturer's protocols. The activity levels of AST and ALT were calculated with normalization to total protein concentration. The histological analysis of liver tissue slides was performed by H&E staining. Tissue imaging was done using an inverted microscopy Axio Observer 7 (Carl Zeiss AG, Oberkochen, Germany) and the analysis was performed by ZEISS Zen 3.9. (Carl Zeiss AG).

### Flow cytometry

For *in vitro* assays, single cell suspensions were obtained from the T cell/tumor cell cocultures of the real-time cytotoxicity assays. For *in vivo* assays, single cell suspensions were prepared from tumors, spleens and peripheral blood. In brief, splenocytes and PBMCs were treated with ACK lysis buffer to remove red blood cells (Life Technologies), while lymphocytes in the tumor cell suspension were isolated using Percoll (Cytiva) density gradient centrifugation. All cells were resuspended in PBS supplemented with 0.5% FBS and 0.02% sodium azide (FACS buffer) before staining.

The cells were stained with viability dye and were treated with human FcR blocking reagent for 15 min each. For extracellular antigen staining, the cells were stained with specific antibodies in FACS buffer for 30 min at 4 °C, and washed with FACS buffer before analysis. For intracellular antigen detection, the cells were stimulated with 50 ng/mL phorbol 12-myristate 13-acetate (PMA) and 500 ng/mL ionomycin in the presence of 10 μg/mL Brefeldin A (BFA) for 4 h at 37 °C with 5% CO_2_. Following extracellular antigen staining, the cells were fixed and permeabilized using Foxp3/transcriptional factor fixation/permeabilization concentrate (eBiosciences) for 30 min at 4 °C. The cells were then stained intracellularly with antibodies diluted with 1x permeabilization buffer for 30 min at 4 °C. The samples were resuspended in FACS buffer and acquired using LSRFortessa^TM^ X20 Cell Analyzer (BD Biosciences, NJ, USA). Data analysis was conducted with FlowJo™ software (Treestar, OR, USA).

### Statistical analysis

Comparisons between two groups were done using Students' unpaired t-test. Multiple comparisons were run using one-Way ANOVA with Bonferroni's post-hoc test. All statistical analysis was done using GraphPad Prism version 10 (GraphPad software, CA, USA). Data were presented as mean±SEM, with p < 0.05 being taken as statistically significant. * p < 0.05, ** p < 0.01 and *** p < 0.001.

## Results

### A high CD137 expression profile in NPC correlates with reduced T cell infiltration

**Figure 1:**
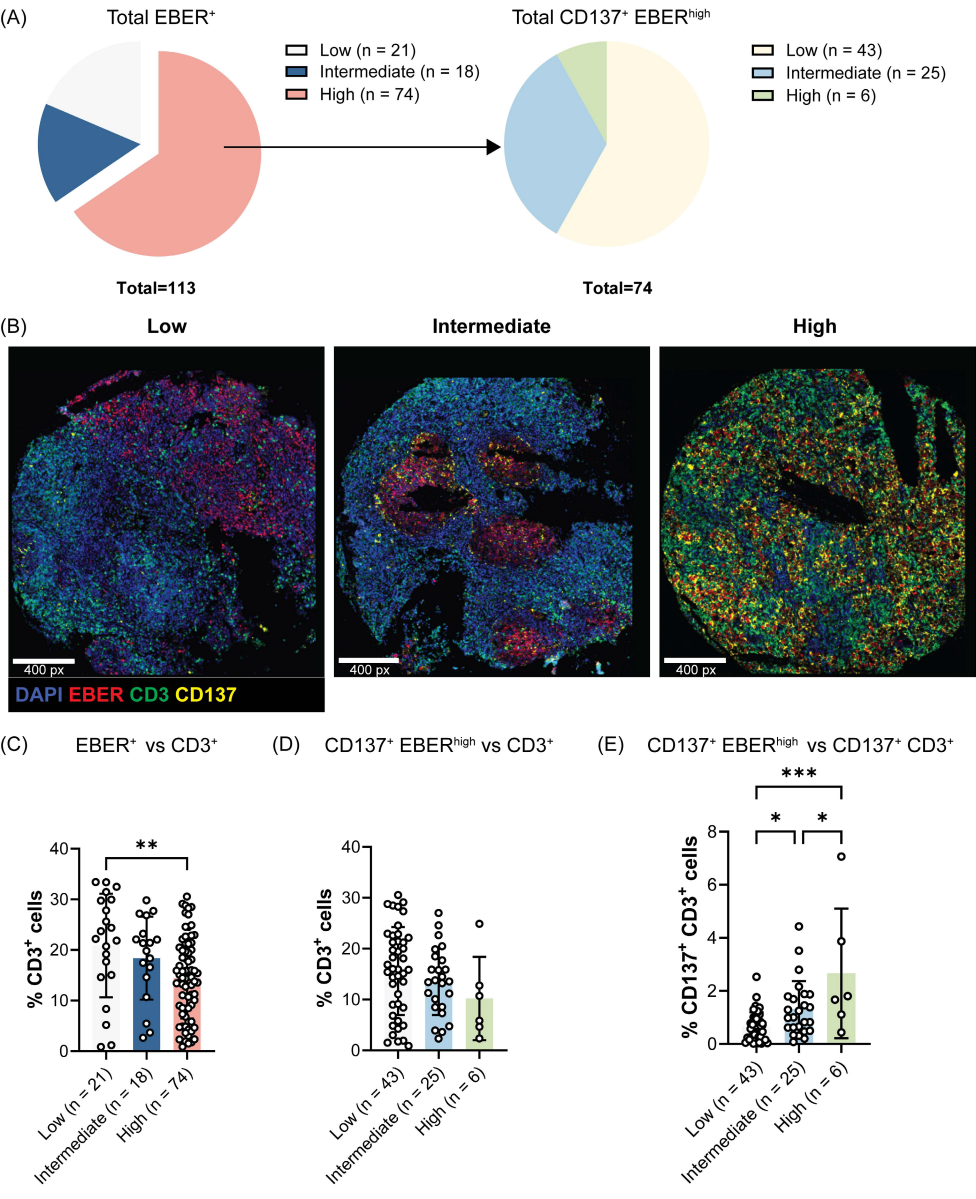
** CD137 expression on EBER^+^ NPC cells negatively associates with T cell tumor infiltration.** Multiplex immunofluorescence staining of a human NPC tissue microarray was conducted to detect EBER, CD3 and CD137 markers. (A) Pie chart illustrating the percentages of tumor cores with low, intermediate and high EBER staining. EBER high cases were further categorized according to the frequency of EBER^+^ NPC cells expressing CD137. (B) Representative images for each frequency level of CD137^+^ EBER^+^ NPC cells are shown. Analysis of the frequency of CD3^+^ T cells across tumor groups with (C) different levels of EBER^+^ NPC cells and (D) of CD137^+^ EBER^+^ NPC cells. (E) The frequency of CD137^+^ CD3^+^ T cells across tumor groups with varying levels of CD137^+^ EBER^+^ NPC cells. Each symbol represents a patient sample. Numbers in the brackets indicate the number of cases. Data are shown as means ± SEM of duplicate wells. * p < 0.05, ** p < 0.01, *** p < 0.001 using one-way ANOVA with Bonferroni's multiple comparison test.

We have previously demonstrated that EBV induces the ectopic expression of CD137 on NPC cells which helps EBV to escape immune surveillance and promotes tumorigenesis in human NPC patients 19. Therefore, we investigated whether CD137 expression on NPC cells influences T cell infiltration into the tumor microenvironment. Of the 132 NPC cases included in the tissue microarray, 113 cases with evaluable staining and sizeable numbers of EBV-encoded RNA (EBER)^+^ NPC cells were analyzed, comprising 77 male and 36 female patients. The cohort had a median age of 48 years (range 10 to 75), with all patients diagnosed at stage 3 disease. The baseline characteristics of the study population are summarized in Table S1. Of the selected 113 cases, 21, 18 and 74 cases exhibited low, intermediate and high frequencies of EBER^+^ NPC cells, respectively (Figure 1A). The 74 tumor cores with high EBER^+^ NPC cells were further stratified into three groups based on the frequency of EBER^+^ NPC cells expressing CD137: low (n = 43), intermediate (n = 25), and high (n = 6) groups. Representative samples of each frequency level of CD137^+^ EBER^+^ NPC cells are shown (Figure 1B).

It was observed that tumor cores with a higher frequency of EBV-infected malignant cells, as indicated by the expression of EBER, were significantly associated with a lower infiltration of CD3^+^ T cells (Figure 1C). In particular, a negative correlation was observed between the frequency of CD137^+^ EBER^+^ NPC cells and tumor-infiltrating CD3^+^ T cells, with the number of CD3^+^ T cells decreasing progressively from tumor cores with low to high levels of CD137^+^ EBER^+^ cells (Figure 1D). Within the CD3^+^ T cell population, CD137-expressing T cells were found at a higher frequency in tumor cores with high CD137^+^ EBER^+^ NPC cells than in tumor cores with low CD137^+^ EBER^+^ NPC cells (Figure 1E).

Previous work by Labiano and colleagues revealed that tumor cells upregulate their expression of soluble CD137 (sCD137) under hypoxic conditions as a mechanism to evade immune detection by neutralizing CD137L on APCs 31. In turn, silencing sCD137 expression in malignant cells promoted T cell-mediated anti-tumor immunity, particularly through CD8^+^ T cells, leading to reduced tumor progression and, in some cases, complete tumor rejection *in vivo*. Supporting the role of CD137 in tumor immune escape, our group has shown that CD137 ectopically expressed by NPC cells can deplete CD137L on APCs via trogocytosis 19.

Therefore, although further validation is required, the upregulation of CD137 expression on NPC cells in the context of abundant CD137^+^ T cells observed in NPC may reflect an immune evasion strategy, wherein NPC-expressed CD137 competitively binds CD137L on APCs to limit costimulatory signaling to T cells. This provided the rationale for engineering rhCD137L-MSNs which bind to CD137 on NPC cells and neutralize it, thus converting the immune-suppressive axis to a T cell-stimulatory signal.

### Conjugation of rhCD137L to MSNs

**Figure 2:**
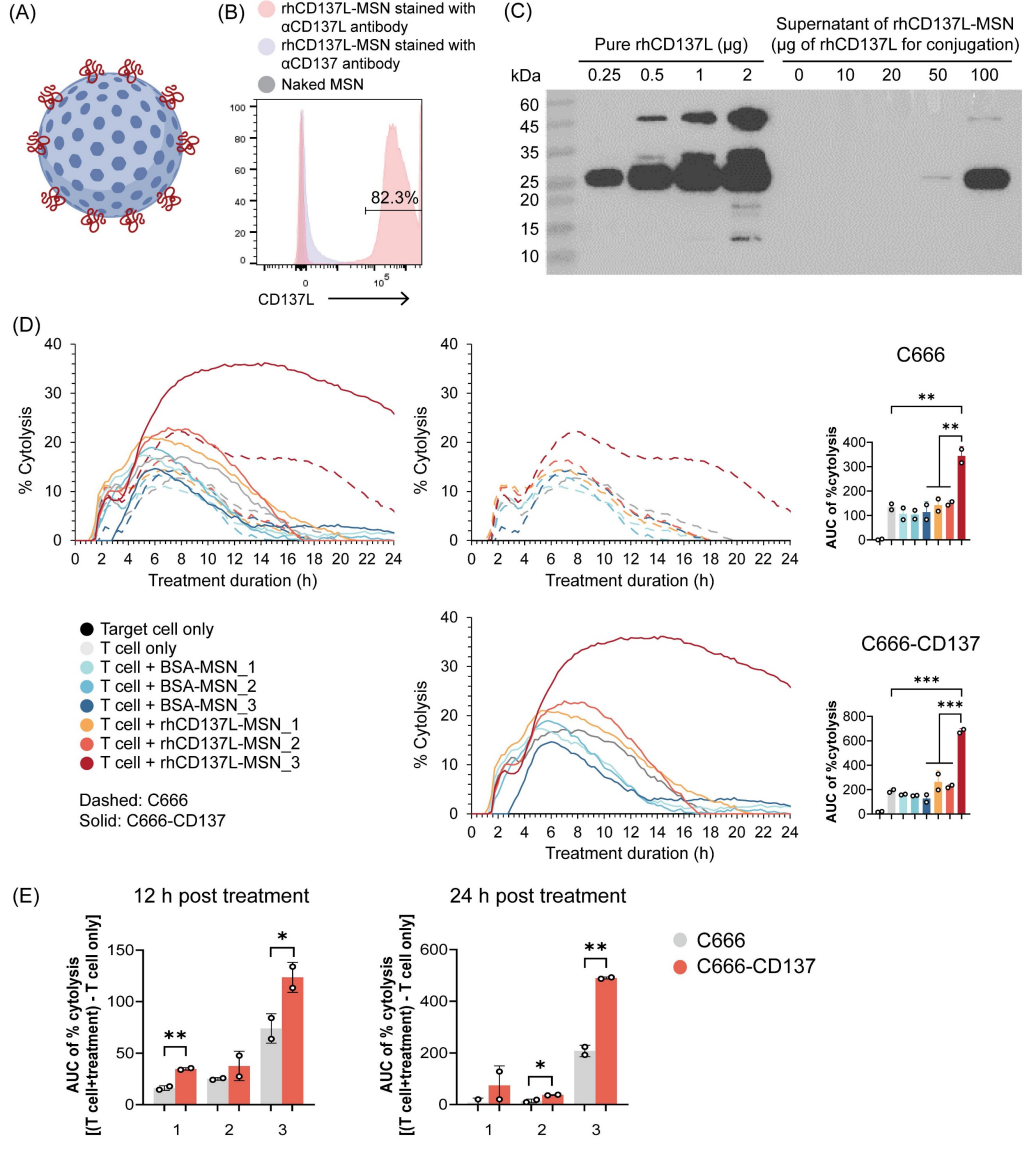
** Generation of rhCD137L-MSN conjugates.** (A) Schematic representation of rhCD137L-MSNs. Blue sphere: MSN, red line: rhCD137L (B) Detection of rhCD137L on MSNs using flow cytometry. Anti-CD137 antibody was included as a negative control for unspecific staining. (C) Optimization of rhCD137L protein amount (0-100 μg) for conjugation onto 1.6x10^10^ MSNs. Equal volumes of supernatants after rhCD137L-MSNs conjugation were used for Western blot analysis to determine the amounts of residual unbound protein. Increasing amounts of free rhCD137L protein were included as reference. Different numbers of MSNs (1: 1.6x10^10^; 2: 3.2x10^10^; 3: 6.4x10^10^) were used to conjugate a fixed amount (20 μg) of rhCD137L protein or BSA. The effects of these rhCD137L-MSNs on T cell-mediated tumor cell cytolysis were assessed using C666 or C666-CD137 cells, with the conjugates administrated at an equal concentration (5 μg/mL) of rhCD137L at 0 h. (D) % cytolysis curves and AUC of % cytolysis over 24 h post treatment. The left line graph combines both cell lines (C666 and C666-CD137) whereas the two graphs on the right are for the individual cell lines. (E) Comparisons of % cytolysis between C666 and C666-CD137 cells over 12 or 24 h post treatment with rhCD137L-MSNs of different MSN numbers. Each symbol represents a technical replicate. Data are shown as means ± SEM of duplicate wells. Data are representative of two independent experiments with T cells of different donors. * p < 0.05, ** p < 0.01, *** p < 0.001 using one-way ANOVA with Bonferroni's multiple comparison test for (D), while using Students' t-test for (C). BSA: bovine serum albumin. MSN: mesoporous silica nanoparticle. AUC: area under the curve.

Multimerization and/or clustering of CD137 agonists enhances their potency 32. To generate multimeric rhCD137L, MSNs of approximately 50 nm diameter were functionalized with positively charged amine groups (-NH_2_), facilitating covalent linkage to the carboxyl groups (-COOH) of rhCD137L through the formation of peptide bonds (Figure 2A). Flow cytometric analysis confirmed the successful synthesis of rhCD137L-MSNs (Figure 2B).

To optimize the loading of rhCD137L molecules to MSNs, a fixed number of MSNs (1.6x10^10^) was mixed with increasing amounts of rhCD137L, ranging from 0 to 100 μg. The amount of unbound rhCD137L present in the supernatant was measured by Western blot analysis (Figure 2C). By subtracting the unbound protein from the initial loading amount, we calculated the overall encapsulation efficiency of rhCD137L to be 99.63% when 100 μg rhCD137L was used as initial loading amount. An increase in loading efficiency was observed as the initial loading amount of rhCD137L decreased, with efficiencies of 99.97% at 50 μg, and 100% at 10 μg and 20 μg. Due to the maximal encapsulation efficiency at the highest rhCD137L quantity, 20 μg was selected as the optimal loading amount for rhCD137L.

However, excessive protein loading onto MSNs may induce protein aggregation, which could compromise the functional integrity of the protein. Indeed, rhCD137L-MSNs optimized for maximal encapsulated efficiency (20 μg rhCD137L + 1.6x10^10^ MSNs) failed to promote effector T cell-mediated killing of the CD137-expressing NPC cell line (C666-CD137) and its CD137-deficient control cells (C666) (Figure 2D). Conversely, increasing the number of MSNs to 6.4x10^10^ during conjugation restored the ability of rhCD137L-MSNs to drive effective T cell-mediated cytotoxicity against both cell lines. Importantly, we excluded the possibility that the enhanced tumor cytolysis was attributed to the increased quantity of MSNs, as control BSA-conjugated MSNs (BSA-MSNs) showed no impact on T cell cytotoxicity. Further evidence of the rhCD137L-MSN (with 6.4x10^10^ MSNs) costimulatory function was demonstrated by its ability to enhance the proliferation of CD137^hi^ CD8^+^ T cells, even beyond their inherently greater proliferative capacity compared to CD137^lo^ counterparts (Figure S1). Given that 6.4x10^10^ MSNs preserved the bioactivity of rhCD137L without inducing non-specific effects, it was thus selected for generating rhCD137L-MSNs used in the subsequent experiments.

### rhCD137L-MSNs drive stronger tumor cytotoxicity than anti-CD137 agonistic antibody-conjugated MSNs *in vitro*

To validate the anti-tumor effect of rhCD137L-MSNs, we compared their ability in enhancing T cell-mediated tumor cytolysis to that of MSNs loaded with anti-CD137 antibodies urelumab and utomilumab, herein referred to as ure-MSNs and uto-MSNs, respectively. The real-time cytotoxicity assay was performed by coculturing T cells with NPC cell lines of both CD137-expressing and CD137-null phenotypes in the presence of rhCD137L-MSNs or anti-CD137 antibody-conjugated MSNs. Given that the antibodies have two CD137 binding domains, while rhCD137L only possesses one, the comparison was done at both equal molar concentrations of receptor binding sites and equal numbers of MSNs to allow a more precise assessment.

**Figure 3:**
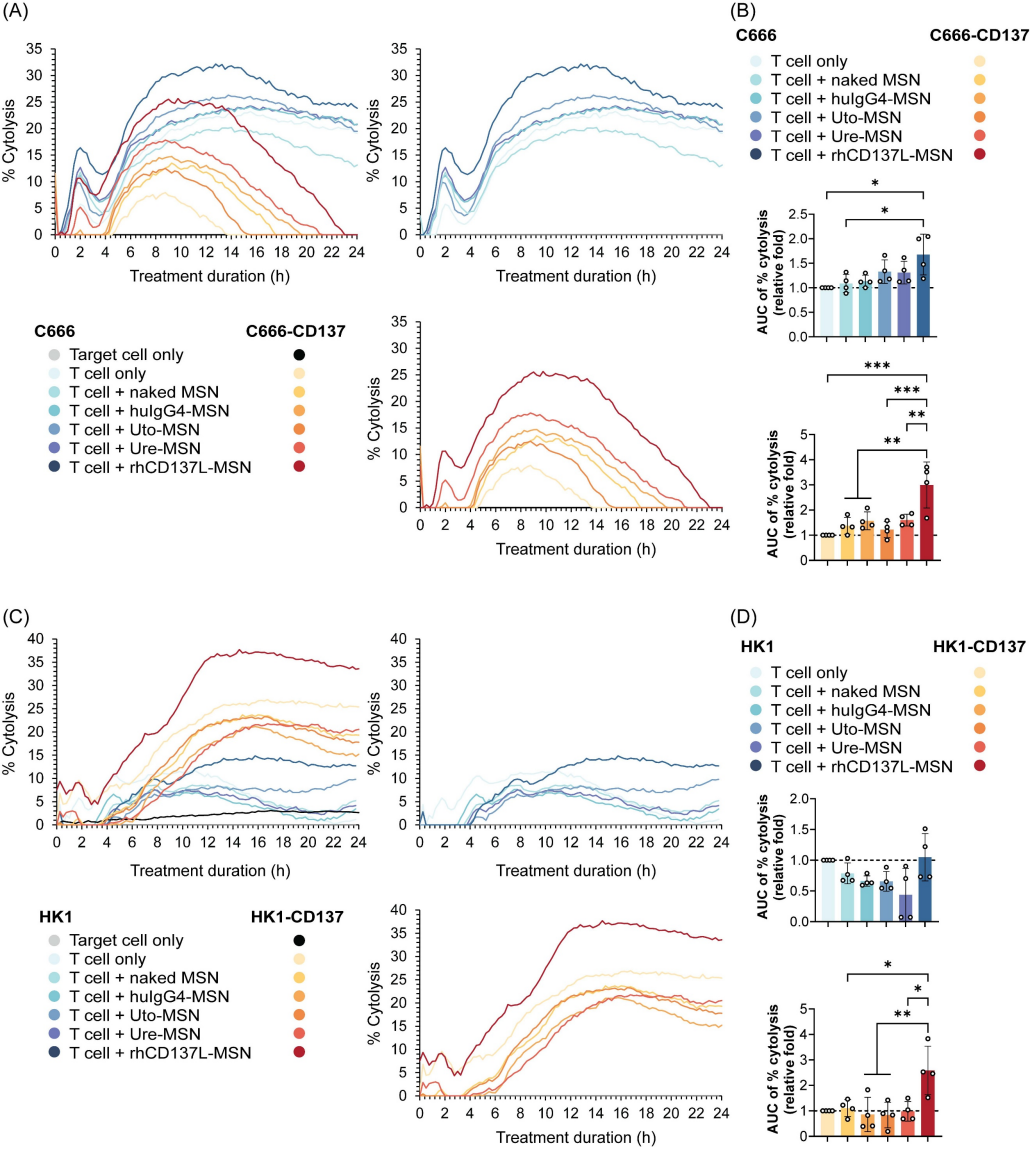
** rhCD137L-MSNs enhance T cell-mediated cytotoxicity against CD137-expressing and -null NPC cell lines.** The T cell stimulatory function of rhCD137L-MSNs was compared to that of anti-CD137 antibodies urelumab (ure) and utomilumab (uto)-conjugated MSNs, at an equal molar concentration of receptor binding sites (197.5 nM). Two groups of NPC cell lines were used as target cells: (A-B) C666 and C666-CD137 and (C-D) HK1 and HK1-CD137. (A & C) % tumor cytolysis induced by indicated treatments over 24 h. The data are representative of four independent experiments with T cells of different donors. (B & D) Normalized ratios (T cell+treatment/T cell only control) for the area under the curve (AUC) of % cytolysis over 24 h post treatment. Each symbol represents a different T cell donor (n = 4). A ratio of 1 (dashed line) indicates no difference between T cell+treatment group and T cell only control. Data are shown as means ± SEM. * p < 0.05, ** p < 0.01, using one-way ANOVA with Bonferroni's multiple comparison test. MSN: mesoporous silica nanoparticle.

As shown in Figure 3A-B, rhCD137L-MSNs exhibited a greater potency than ure-MSNs and uto-MSNs in promoting T cell-mediated cytotoxicity of both C666 and C666-CD137 cells. Ure-MSNs and uto-MSNs, however, showed little to no enhancement of T cell killing activity, compared to the negative controls huIgG4-MSNs and naked MSNs. Notably, the extent of tumor cell death induced by rhCD137L-MSNs was significantly higher in C666-CD137 cells than in C666 cells. These results were confirmed using another set of NPC cell lines: HK1 and HK1-CD137 cells (Figure 3C-D).

We also excluded the possibility that MSN conjugation compromised antibody efficacy by comparing the effect of rhCD137L-MSNs with those of unconjugated urelumab and utomilumab. Consistent with our previous findings, rhCD137L-MSNs demonstrated superior efficacy over both urelumab and utomilumab in boosting T cell cytotoxicity against C666 cells and even more so against C666-CD137 cells, at an equal molar concentration of receptor binding sites (Figure S2A-B).

In addition, we also explored StrepTactin® XT 4Flow® resin beads (referred to as ST) as an alternative multimerization platform to complement the findings obtained using MSNs. A TwinStrep tag sequence was incorporated to the N-terminal end of rhCD137L ectodomain to enable the binding of rhCD137L protein to ST beads (Figure S3A). Conjugation of 0.64 μM ST beads to 312.5 nM rhCD137L facilitated the formation of trimeric and tetrameric rhCD137L to the greatest extent compared to other ST bead concentrations, although the majority of rhCD137L existed in a dimeric form (Figure S3B). Using rhCD137L-ST complexes, we obtained similar results as with rhCD137L-MSNs, in which rhCD137L-ST promoted a greater cytolysis of both CD137-expressing (C666-CD137 and HK1-CD137) and CD137-deficient (C666 and HK1) NPC cell lines, than unconjugated urelumab and utomilumab, when added at an equal protein concentration (Figure S3C-D). This effect was significantly greater in C666-CD137 and HK1-CD137 cells than in their respective CD137-deficient control cells. Since MSNs were smaller in size and therefore more suitable for *in vivo* studies, we conducted all subsequent experiments with MSNs.

### Treatment with rhCD137L-MSNs enhances T cell activation and expression of effector molecules

**Figure 4:**
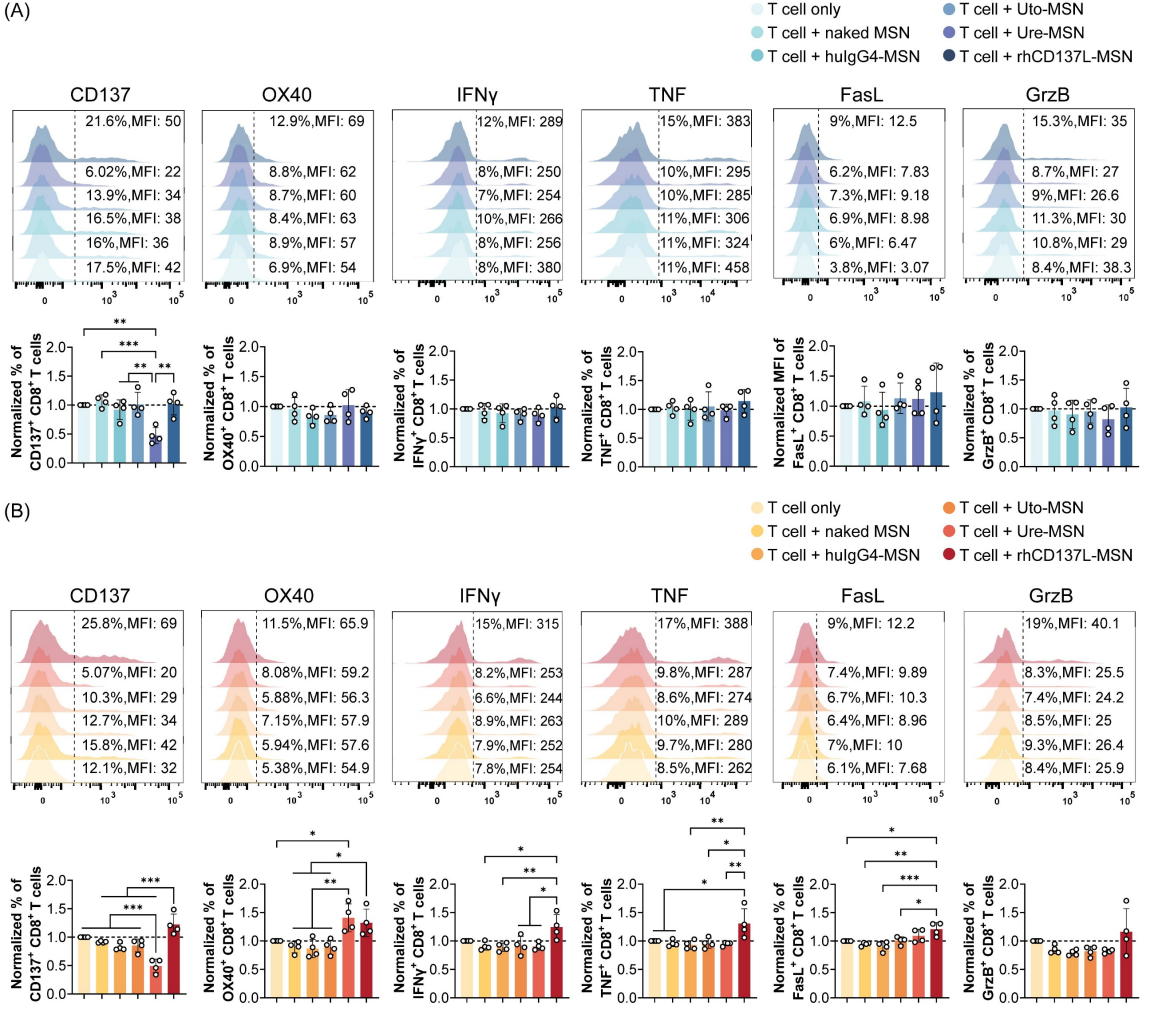
** Treatment with rhCD137L-MSNs significantly enhances the activation and effector functions of CD8^+^ T cells that were cocultured with C666-CD137 cells.** The expression levels of CD137, OX40, IFNγ, TNF, FasL and granzyme B (GrzB) in CD8^+^ T cells that were cocultured with (A) C666 and (B) C666-CD137 cells, were analyzed using flow cytometry. The expression levels of these markers in each treatment group were normalized to their levels in the T cell only treatment group. The results were obtained from T cells of four different donors, with each symbol representing an individual T cell donor (n = 4). A ratio of 1 (dashed line) indicates no difference between T cell+treatment group and T cell only control. Data are shown as means ± SEM. * p < 0.05, ** p < 0.01, *** p < 0.001 using one-way ANOVA with Bonferroni's multiple comparison test. Uto: utomilumab. Ure: urelumab. MSN: mesoporous silica nanoparticle.

We next assessed the costimulatory function of rhCD137L-MSNs on T cells that were cocultured with tumor cells. Flow cytometry analysis showed that, when cocultured with C666 cells, CD8^+^ and CD4^+^ T cells exhibited no significant changes in their expression of CD137, OX40, IFNγ, TNF, granzyme B and FasL across different treatment conditions (Figure 4A & S4A). However, in the presence of C666-CD137 cells, rhCD137L-MSNs significantly promoted the frequency of CD8^+^ T cells expressing CD137, OX40, IFNγ or TNF, whereas ure-MSNs promoted only OX40 expression while reducing CD137 level, probably via internalization (Figure 4B). Furthermore, rhCD137L-MSNs also increased the expression of FasL and granzyme B by CD8^+^ T cells, despite the overall low frequencies of these cells. Likewise, CD4^+^ T cells gained a higher activation status with rhCD137L-MSN treatment, as evidenced by marked elevated levels of CD137, OX40 and IFNγ, in addition to a marginal increase in TNF, FasL and granzyme B expression (Figure S4B).

In coculture with HK1 cells, CD8^+^ T cells displayed a trend towards increased TNFα expression in response to rhCD137L-MSNs, while CD4^+^ T cells exhibited an increase in FasL expression following treatment with rhCD137L-MSNs (Figure S5A). Conversely, rhCD137L-MSNs induced a significant upregulation of FasL expression on both CD8^+^ and CD4^+^ T cells cocultured with HK1-CD137 cells, an effect not seen with ure-MSN and uto-MSN treatment, when compared to T cell treatment alone (Figure S5B). No significant changes were observed in the expression of CD137, IFNγ, and granzyme B for CD8^+^ and CD4^+^ T cells cocultured with both HK1 and HK1-CD137 cells across all treatment conditions. Altogether, rhCD137L-MSNs show a stronger T cell costimulatory potential than ure-MSNs and uto-MSNs, particularly in the presence of CD137-expressing cancer cells.

In addition to T cells, we examined the effects of rhCD137L-MSNs on CD137-expressing NPC cells. Consistent with our previous findings using agonistic CD137 antibodies, treatment with rhCD137L-MSNs promoted the production of tumor-associated cytokines, specifically IL-6 and IL-8 by C666-CD137 cells, and IL-8 by HK1-CD137 cells (Figure S6A-B) 19. Comparable effects were also observed with ure-MSNs, whereas uto-MSNs demonstrated little to no activity. No cytokine induction was detected in CD137-deficient C666 and HK1 cells across different treatments. These findings indicate that rhCD137L-MSNs could support NPC tumorigenesis in terms of cytokine production. Despite this, significant tumor cell death was observed following rhCD137L-MSN treatment. This suggests that the net effect of rhCD137L-MSNs remains anti-tumoral, primarily through - but not limited to - the induction of effector T cell-mediated tumor cell death which is likely to precede the production of tumor-associated cytokines.

### rhCD137L-MSNs control tumor growth *in vivo* more effectively than ure-MSNs

To explore the *in vivo* therapeutic efficacy of rhCD137L-MSNs in a clinically relevant setting, we employed PBMC-humanized NOD-scid (JInx) mice, bearing NPC of CD137-expressing (C666-CD137 or HK1-CD137) and -deficient (C666 or HK1) phenotype on the different flanks. These humanized NPC models were employed due to the human-specific tropism of EBV, which does not infect mice and precludes the use of immunocompetent or syngeneic NPC models, thereby preserving the translational significance of our findings.

For the initial *in vivo* biodistribution assessment, a single intravenous dose (10 mg/kg) of rhCD137L-MSNs was administered into C666/C666-CD137-bearing humanized mice, followed by bioluminescent imaging 48 hours post-injection. Compared to naked MSNs, rhCD137L-MSNs demonstrated enhanced migration to C666 and C666-CD137 tumors (Figure S7A-B). Notably, the infiltration of rhCD137L-MSNs into C666-CD137 tumors was more efficient than into CD137-deficient C666 tumors. These observations were further validated through ex vivo tumor imaging (Figure S7C-D). Infiltration levels of naked MSNs and rhCD137L-MSNs were comparable across non-tumor organs (Figure S7E-F). These findings suggest that surface-decoration of MSNs with rhCD137L potentially facilitates their engagement with T cells, and drives MSN-T cell localization within tumors. Additional tumor-associated CD137 expression further enables the targeted delivery of MSNs and/or MSN-T cells to tumors. As such, rhCD137L-MSNs were hypothesized to exert a tumor-targeted therapeutic effect.

**Figure 5:**
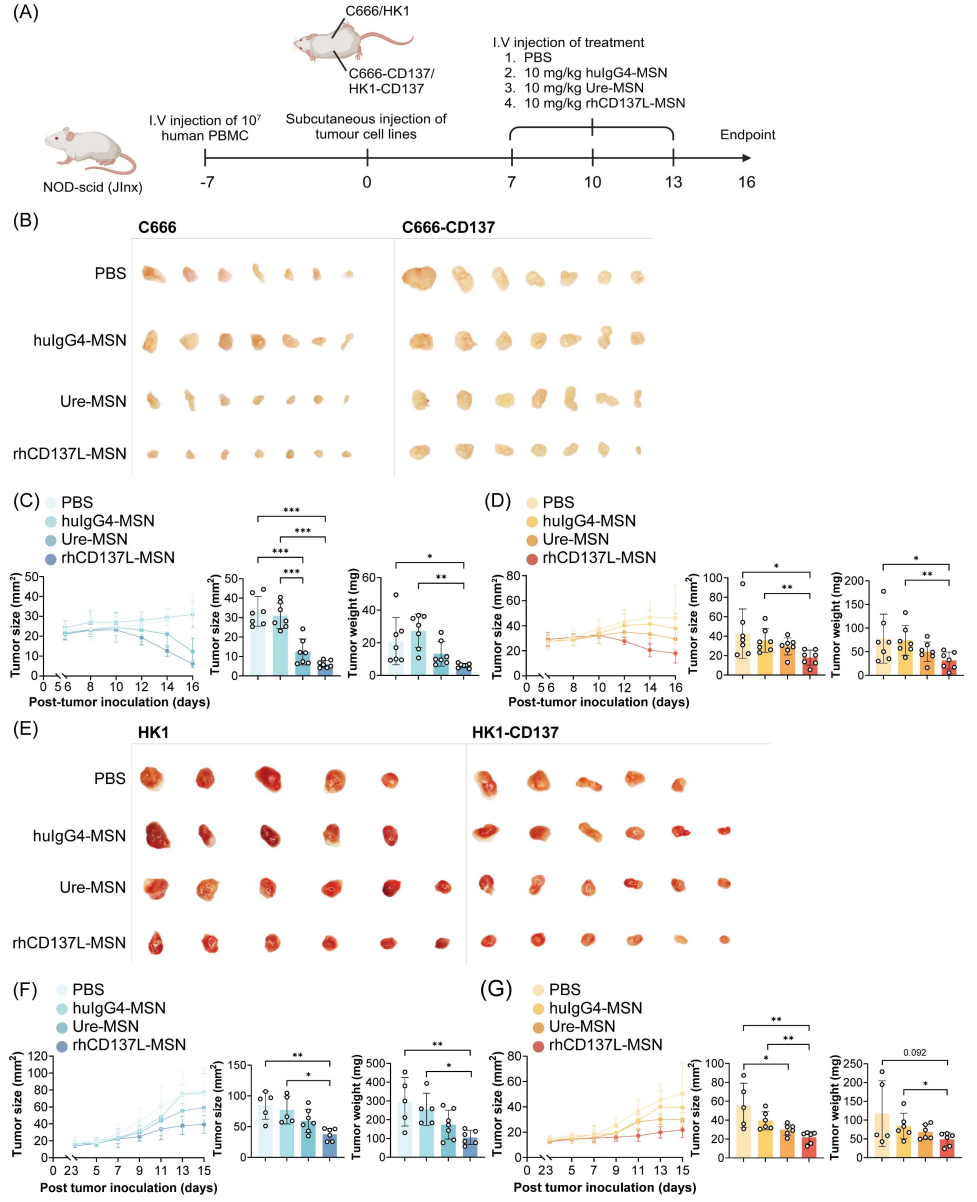
** rhCD137L-MSNs efficiently control growth of CD137-expressing and CD137-null NPC in humanized mice.** (A) NOD-scid (JInx) mice were injected intravenously with 10^7^ human PBMC to establish a humanized murine model. One week later, the mice were subcutaneously injected with 10^6^ CD137-expressing (C666-CD137 and HK1-CD137) and CD137-null (C666 and HK1) NPC cells into the left and right flanks, respectively. When tumors had grown to a palpable size, rhCD137L-MSNs or ure-MSNs were administered intravenously at a dose of 10 mg/kg of body weight for a total of three doses at three-day intervals. PBS and huIgG4-MSN treatments served as negative controls. Images of (B) C666 and C666-CD137 tumors and (E) HK1 and HK1-CD137 tumors from mice of the different treatment groups. Tumor growth curves, tumor sizes and weights at endpoint for (C) C666, (D) C666-CD137, (F) HK1 and (G) HK1-CD137. Each symbol represents one mouse (n = 5-6). Data are shown as means ± SEM. Data are representative of two independent experiments. * p < 0.05, ** p < 0.01, *** p < 0.001 using one-way ANOVA with Bonferroni's multiple comparison test. PBS: phosphate buffered saline. Ure: urelumab. MSN: mesoporous silica nanoparticle.

Subsequent therapeutic evaluation involved three intravenous doses of rhCD137L-MSNs or ure-MSNs (10 mg/kg), each given at three days intervals (Figure 5A). Here, urelumab was selected over utomilumab due to its superior potency as a CD137 agonist. Compared to the huIgG4-MSNs and PBS treatment which served as negative controls, treatment with rhCD137L-MSNs and ure-MSNs profoundly and significantly reduced the growth of both C666 and C666-CD137 tumors, with rhCD137L-MSNs inducing a further tumor reduction than ure-MSNs (Figure 5B-D). Within each tumor type, tumor sizes and weights were significantly smaller in the rhCD137L-MSN groups than the huIgG4-MSNs and PBS treatment groups and were on average smaller than those in the ure-MSN groups. Nonetheless, C666-CD137 tumors were generally larger than C666 tumors, regardless of the different treatments, due to the faster proliferation rate of C666-CD137 cells compared to C666 cells, as shown in the *in vitro* proliferation assay (Figure S8A). Comparable therapeutic effects were obtained with mice bearing HK1 and HK1-CD137 tumors, which exhibited similar proliferation rate *in vitro* (Figure 5E-G & S8B). Specifically, treatment with rhCD137L-MSNs and ure-MSNs delayed tumor growth, with rhCD137L-MSNs exhibiting a stronger therapeutic effect than ure-MSNs.

### rhCD137L-MSNs enhance tumor infiltration of effector memory CD8^+^ T cells

**Figure 6:**
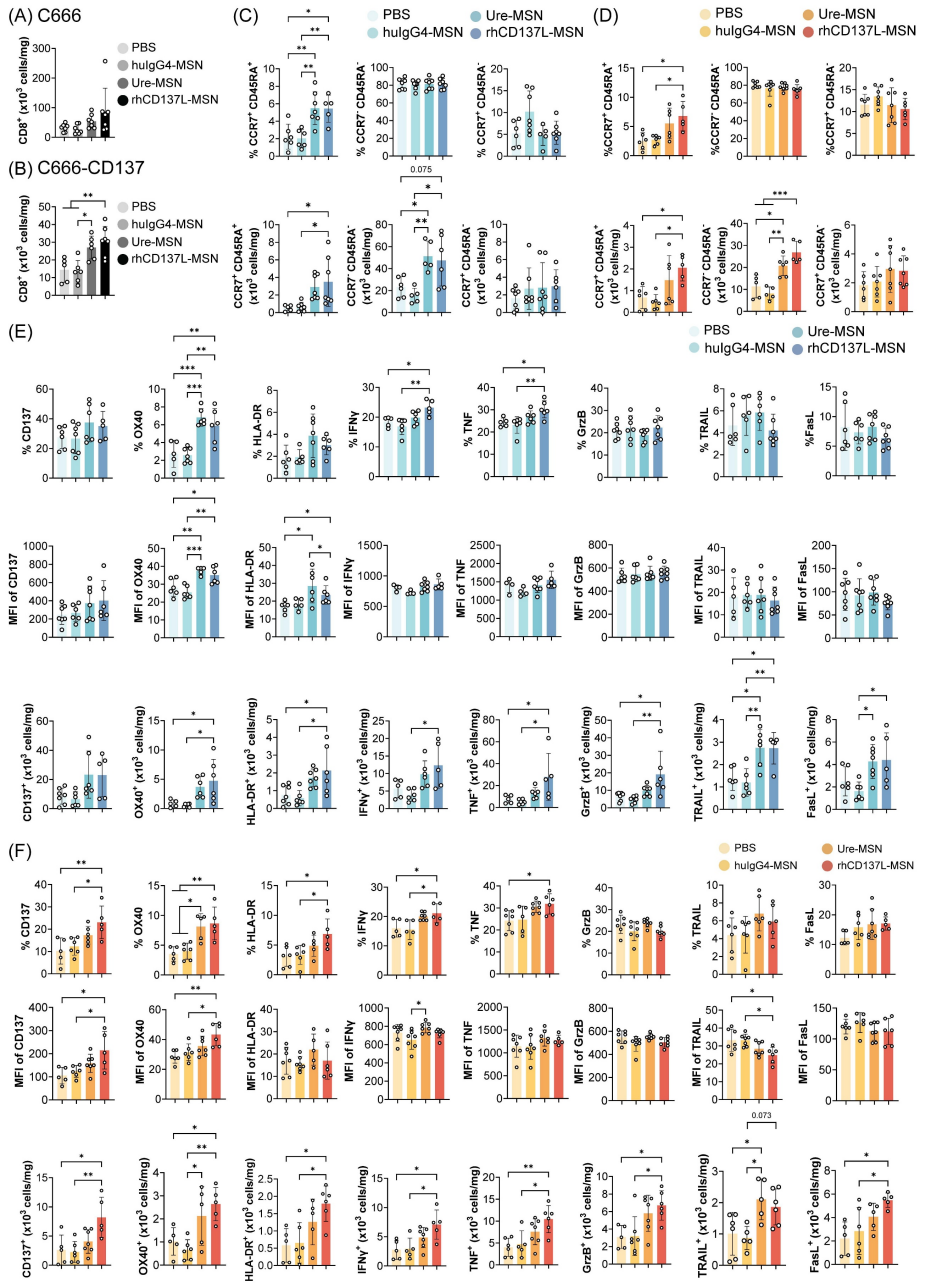
** Increased tumor infiltration of activated and polyfunctional CD8^+^ T cells upon rhCD137L-MSN treatment.** Number of CD8^+^ T cells in (A) C666 and (B) C666-CD137 tumors of the different treatment groups. CD8^+^ T cells in the state of naïve (CCR7^+^ CD45RA^+^), effector memory (CCR7^-^ CD45RA^-^), and central memory (CCR7^+^ CD45RA^-^) were examined in (C) C666 and (D) C666-CD137 tumors. The data are shown as % population and absolute numbers of cells. Expression levels of CD137, OX40, HLA-DR, IFNγ, TNF, granzyme B (GrzB), TRAIL and FasL of tumor-infiltrating CD8^+^ T cells in (E) C666 and (F) C666-CD137 tumors were analyzed in terms of % population, mean fluorescence intensity (MFI), and absolute numbers of cells. Each symbol represents one mouse (n = 5-6). All data are shown as means ± SEM. Data are representative of two independent experiments. * p < 0.05, ** p < 0.01, *** p < 0.001 using one-way ANOVA with Bonferroni's multiple comparison test. PBS: phosphate buffered saline. Ure: urelumab. MSN: mesoporous silica nanoparticle.

To elucidate the mechanisms driving the anti-tumor effect of rhCD137L-MSNs, we examined the infiltration of CD8^+^ T cells into C666 and C666-CD137 tumors. With rhCD137L-MSN treatment, an increase in CD8^+^ T cell infiltration into both C666 and C666-CD137 tumors was observed (Figure 6A-B). Similar effects were induced by ure-MSNs. Notably, the overall frequency of CD8^+^ T cells in C666-CD137 tumors was lower than in C666 tumors, regardless of treatment groups. Among the tumor infiltrating CD8^+^ T cells, rhCD137L-MSNs and ure-MSNs substantially expanded the proportion of CCR7^-^ CD45RA^-^ effector memory T cells and, to a lesser extent, of CCR7^+^ CD45RA^+^ naïve T cells (Figure 6C-D). The population of CCR7^+^ CD45RA^-^ central memory CD8^+^ T cells remained unchanged across treatments.

In spleens, rhCD137L-MSNs also significantly increased the frequency of CD8^+^ T cells, which was mainly due to a robust expansion of the effector memory subset, an effect not observed with ure-MSNs (Figure S9A & C). While the total number of CD8^+^ T cells in peripheral blood was unchanged across all treatment groups, a higher proportion of effector memory subset was induced by rhCD137L-, ure- and huIgG4-MSN treatments compared to the PBS group (Figure S9B & D).

Likewise, in HK1 and HK1-CD137 tumors, rhCD137L-MSNs enhanced CD8^+^ T cell infiltration, especially into the latter, with a significant proportion of these cells exhibiting a CCR7^-^ CD45RA^-^ effector memory phenotype as compared to treatments with huIgG4-MSNs and PBS (Figure S10A-D). In contrast, ure-MSNs did not promote tumor infiltration of CD8^+^ T cells but demonstrated comparable effectiveness in driving the expansion of effector memory CD8^+^ T cells.

### rhCD137L-MSNs promote CD8^+^ T cell activation and functions in the tumor microenvironment

Consistent with potent T cell costimulation observed *in vitro* (Figure 4 & S4), rhCD137L-MSNs induced elevated levels of CD137, OX40, HLA-DR, IFNγ and TNFα in CD8^+^ T cells in both C666 and C666-CD137 tumors (Figure 6E-F). The increase in these markers was observable in terms of population percentage, MFI and cell number. Although there were no changes in the expression levels of granzyme B, TRAIL and FasL, the overall increase in the tumor-infiltrating CD8^+^ T cell subset, induced by rhCD137L-MSNs and ure-MSNs, resulted in a proportional increase in the number of CD8^+^ T cells expressing granzyme B, TRAIL and FasL, in both C666 and C666-CD137 tumors. The costimulatory function of rhCD137L-MSNs was also evident in CD8^+^ T cells derived from HK1 and HK1-CD137 tumors, as indicated by their elevated expression of CD137, OX40, IFNγ, TNF, granzyme B and TRAIL, compared to CD8^+^ T cells from other treatment groups (Figure S10E-F).

In C666/C666-CD137 tumor-bearing, humanized mice, analysis of splenic CD8^+^ T cells revealed that rhCD137L-MSNs modestly enhanced the expression of granzyme B, while marginally reducing the levels of OX40, HLA-DR, TRAIL and FasL, compared to other treatment groups (Figure S9E). In terms of cell numbers, a greater CD8^+^ T cell subset expressing IFNγ, TNFα and granzyme B was observed. On the other hand, the overall levels of CD137, OX40 and HLA-DR in peripheral CD8^+^ T cells were extremely low (Figure S9F). A minimal increase in the expression of CD137, OX40 and FasL was observed in peripheral CD8^+^ T cells following rhCD137L-MSN treatment, while both rhCD137L-MSNs and ure-MSNs induced a decline in IFNγ, TNFα and granzyme B levels. Collectively, these results suggest that rhCD137L-MSNs enhance the activation and functions of tumor CD8^+^ T cells.

### rhCD137L-MSNs exhibit a superior safety profile to ure-MSNs

**Figure 7:**
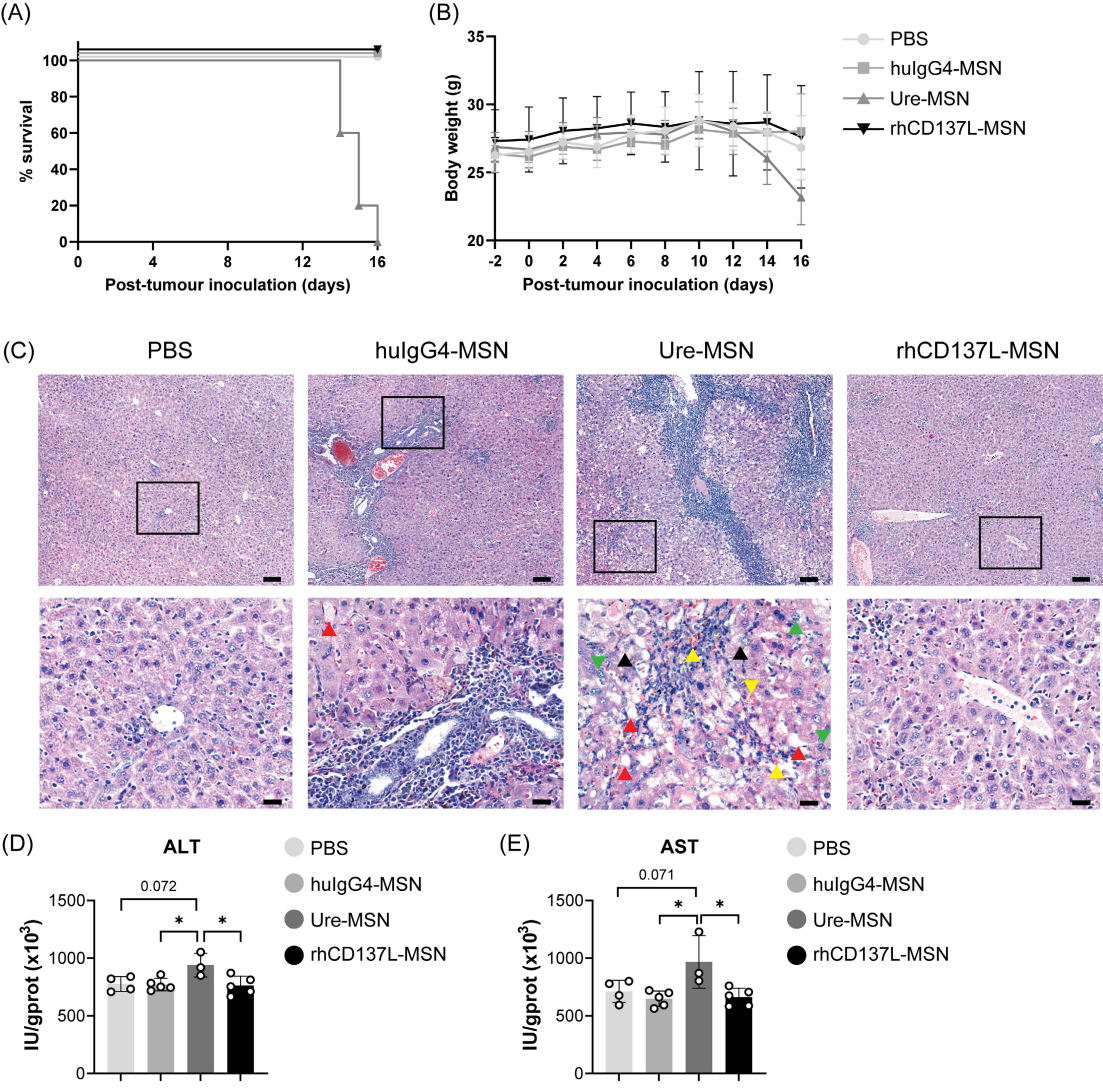
** Ure-MSNs cause severe liver toxicity in NPC-bearing humanized mice.** PBMC-humanized NOD-scid (JInx) mice bearing C666 and C666-CD137 tumors on their two flanks were examined for their (A) survival and (B) body weight changes over time under different treatment conditions: PBS, huIgG4-MSNs, ure-MSNs and rhCD137L-MSNs. (C) Representative H&E-stained liver sections of mice in different treatment groups. The upper panels show the images taken at 10x objective. Scale bar, 100 μm. The bottom panels show the magnified images of the area highlighted in their upper panel images. Green arrow: mitotic figure. Yellow arrow: spreading of erythrocytes to liver tissues. Red arrow: macrovesicular steatosis. Black arrow: ballooning degeneration. Scale bar, 20 μm. Liver (D) alanine aminotransferase (ALT) and (E) aspartate aminotransferase (AST) levels in response to different treatment conditions. Each symbol represents one mouse (n = 3-5). Data are shown as means ± SEM. Data are representative of two independent experiments. Numbers above the brackets indicate p-values. * p < 0.05 using one-way ANOVA with Bonferroni's multiple comparison test. PBS: phosphate buffered saline. Ure: urelumab. MSN: mesoporous silica nanoparticle.

As shown in Figure 7A-B, treatment with rhCD137L-MSNs did not affect the survival and body weight of the mice. However, ure-MSN treatment induced a reduction in the body weight of the mice starting from day 10, and it resulted in all mice either dying or reaching their endpoint (> 20% body weight loss) by day 16.

Given that urelumab has been previously reported to cause severe chronic liver inflammation and hepatocyte damage in preclinical models and human patients 15, 33, the mortality and significant body weight loss observed in mice receiving ure-MSN treatment could have potentially resulted from hepatoxicity. Indeed, histological analysis of livers of mice treated with ure-MSNs displayed pronounced pathological changes compared to those mice in other treatment groups (Figure 7C). These changes were characterized by extensive hepatocyte necrosis and hyperplasia, cellular edema, steatosis and the disorganization of hepatic cord. In contrast, mice treated with rhCD137L-MSNs exhibited liver histological features comparable to mice with PBS treatment, with only slight unevenness in hepatocyte cell size. Surprisingly, huIgG4-MSN treatment resulted in moderate liver damage, featuring marked hepatocyte edema, mild steatosis and minimal hyperplasia. The severe liver damage induced by ure-MSNs was further supported by the significantly elevated levels of ALT and AST in liver tissues (Figure 7D-E). The data indicates that rhCD137L-MSNs have a more favorable safety profile than ure-MSNs.

## Discussion

CD137-based immunotherapies have considerable potential in activating and reinvigorating tumor-specific T cell responses. However, achieving a balance between therapeutic efficacy and safety has been a challenge. Since Fc-mediated enhancement of CD137 agonism has been implicated in the off-tumor toxicities of anti-CD137 antibodies, CD137 agonists with robust potency independent of Fc receptor crosslinking emerge as superior alternatives 34. Here, we developed a novel CD137 agonist by multimerizing rhCD137L using MSNs. These multimeric rhCD137L-MSNs incorporate the two advantages of having potent T cell costimulatory activity in the tumor and minimizing liver damage that can be associated with conventional antibody-based agonists. Moreover, we demonstrate for the first time that co-targeting CD137 on tumor cells with rhCD137L-MSNs enhances T cell-mediated cytotoxicity against CD137-expressing tumor cells.

Akin to most members of the TNFSF, the homotrimeric form of CD137L represents the basic signaling unit required for the formation of a functionally active CD137 receptor cluster, which is essential for initiating downstream TRAF-mediated signaling pathways that drive T cell activation 35. This importance of the trimeric structure of CD137L has been supported by multiple lines of evidence, showing that recombinant CD137L, which predominantly exists in the monomeric and dimeric form, lacks the ability to trigger CD137 signaling 36, 37. In accordance with these earlier findings, our data demonstrate that rhCD137L oligomerization using either MSNs or ST beads endows it with a potent costimulatory activity for augmenting the cytotoxicity of T cells. In addition, compared to the anti-CD137 agonistic antibody urelumab, either unconjugated or conjugated to MSNs, rhCD137L-MSNs display a stronger capacity to functionally engage CD137, as reflected by enhanced T cell functions and improved tumor control both *in vitro* (at an equal molar concentration) and *in vivo* (at an equal mass concentration). The superior CD137 agonism triggered by rhCD137L-MSNs may be attributed to the formation of durable CD137 clusters on the surface of T cells, whereas ure-MSNs tend to induce the internalization of CD137 upon binding. Hence, the increased level of CD137 on T cells following *in vitro* treatment with rhCD137L-MSNs, in contrast with its downregulation by ure-MSNs might explain the increased costimulation induced by rhCD137L-MSNs.

In addition, CD8^+^ T cells stimulated with rhCD137L-MSNs show enhanced expression of IFNγ, TNF and to a limited extent, FasL. IFNγ can promote MHC class I expression in target cells and the post-proteasomal trimming of antigen epitope precursors to mature epitopes that fit the binding sites of MHC class I 38-40. As such, this enhances the antigen-specific recognition and killing by target cells by CD8^+^ T cells. Moreover, IFNγ has been reported to induce growth arrest of target cells either independently via the inhibition of the cyclin-dependent kinases, or in combination with TNF through the induction of cellular senescence 41. Of note, CD8^+^ T cell-derived IFNγ can exert their effects not only on target cells that are in direct contact, but also on bystander cells at far distances 42, 43. These IFNγ-mediated anti-tumor activities could potentially contribute to the increased NPC cell death induced by rhCD137L-MSNs, which may be further enhanced by the additive effects of TNF and FasL. Additionally, it was also observed that rhCD137L-MSN treatment results in a higher frequency of tumor-infiltrating CD8^+^ T cells in both CD137-expressing and -deficient NPC. The accumulation of tumor CD8^+^ T cells may be attributed to their increased recruitment to the tumor sites by both IFNγ and TNF, although the potential contribution of enhanced T cell survival and proliferation driven by these cytokines remain a possibility 42, 44. More importantly, our data reveal that a high frequency of EBER^+^ NPC cells, particularly CD137-expressing EBER^+^ NPC cells, is associated with low T cell infiltration, pointing to the suppressing effect of NPC-derived CD137 on T cell-mediated anti-tumor immunity, which consists in the downregulation of CD137L on APC 19. Therefore, supplementing CD137L in the form of rhCD137L-MSNs is able to reverse NPC-mediated T cell suppression and to reshape the NPC immune landscape.

Beyond their costimulatory potency, the toxicity associated with CD137 agonistic antibodies has been a major concern. In preclinical murine models and clinical studies, administration of IgG4-based anti-CD137 antibodies (i.e. urelumab) has resulted in non-specific immune activation, especially in the liver due to Fc-mediated CD137 hyper-crosslinking upon binding to FcγRIIb that is highly expressed by Kupffer cells and sinusoidal endothelial cells in the liver 15, 45-47. Consistent with previous findings, we demonstrate that treatment with ure-MSNs causes severe liver inflammation and damage, along with dramatic body weight loss and increased mortality in mice. In contrast, rhCD137L-MSNs exhibit minimal hepatotoxicity, with liver histology comparable to that in PBS-treated controls, highlighting its favorable safety profile.

Although rhCD137L-MSNs promote tumor cytolysis *in vitro* regardless of CD137 expression by target cells, the extent of killing is substantially higher when tumor cells express CD137. This indicates that rhCD137L-MSNs engage additional mechanisms dependent on the CD137 expression by target cells. First, CD137 on tumor cells likely acts as a platform for immobilizing rhCD137L-MSNs, which in turn supports the formation of dense, hyper-clustered CD137 signaling complexes in T cells at the points of contact with CD137-expressing tumor cells. The necessity of CD137 hyper-crosslinking for inducing strong CD137 signaling has been documented by several multi-specific antibodies that co-target TAAs (i.e. human epidermal growth factor receptor 2 (HER2), epidermal growth factor receptor (EGFR)) or co-modulatory molecules (i.e. PD-L1) as anchoring points 48-50. Second, rhCD137L-MSNs may function as a bridge to shorten the spatial distance between T cells and CD137-expressing tumor cells thus allowing optimal T cell activation. Clinical studies have revealed that a high density of cytotoxic effector cells in close proximity with tumor cells is strongly correlated with enhanced immune cell cytotoxicity against the malignant cells, as well as improved therapeutic responsiveness to immune checkpoint blockade treatment 51-54. Hence, these two mechanisms, either independently and/or in combination, are likely the basis or contributing to the stronger costimulatory potential of rhCD137L-MSNs in the presence of CD137-expressing tumor cells.

Our data support existing studies on the multimerization of CD137L. Multimeric murine CD137L, generated by fusing the CD137L ectodomain to a modified streptavidin core (SA-CD137L), effectively establishes long-term T cell memory responses against tumor challenges and post-surgical recurrences 55. Likewise, the introduction of human collagen XVIII trimerization domain endows human CD137L (s4-1BBL-TriXVIII) with potent costimulatory function on T cell activation and proliferation. Following the administration of oncolytic measles viruses encoding s4-1BBL-TriXVIII, significant tumor regression and prolonged survival were achieved in a CD34^+^ cell-humanized colon cancer model 37. Building upon these foundational works, our study advances CD137L multimerization by employing MSNs, which offer several therapeutic advantages. Unlike conventional multimeric CD137L which functions as an individual entity, a single rhCD137L-MSN can provide numerous CD137L oligomers to a T cell at the point of contact, driving multi-CD137 trimer clustering and inter-CD137 trimer bridging to establish a functional signalosome of greater magnitude. Moreover, using MSNs enhances versatility, as other immunomodulatory molecules can be conjugated together with CD137L for co-delivery to achieve synergistic effects.

Substantial evidence has revealed that combining immune checkpoint inhibitors (ICIs) with CD137 agonists maximizes tumor clearance and control in preclinical models. Sanmamed and team demonstrated that combinatorial treatment with urelumab and nivolumab (anti-human PD-1 antibody) enhances CD8^+^ T cell-mediated anti-tumor responses in xenograft models of human colon and gastric carcinoma 56. The therapeutic impact of ICIs and CD137 agonists is further potentiated when delivered as a single entity 57. IBI319, an anti-PD-1/CD137 bispecific humanized IgG1 antibody induces superior tumor regression compared to the combination of separate anti-PD-1 and anti-CD137 antibodies 58. Consistent findings were also observed using adenovirus co-expressing soluble PD-1/CD137L fusion protein and anti-PD-L1/CD137 bispecific humanized IgG1 antibody (GEN1046) 59, 60. These reported dual-targeting therapeutic agents collectively provide a compelling incentive for future development of MSNs co-functionalized with rhCD137L and ICIs. Beyond that, TAAs such as fibroblast activation protein (FAP), and EGFR could also be partnered with CD137 agonists on a single MSN platform to ensure tumor-specific T cell activation, as supported by earlier studies 49, 61.

Nevertheless, the application of MSNs as nanocarriers hinges on demonstrating biocompatibility and acceptable safety profile in specific therapeutic settings. MSNs have been reported to induce oxidative stress and membranolysis, with the severity of these cytotoxic effects dictated primarily by their physical and physiochemical properties 62-64. Therefore, while MSNs used in this study did not provoke intolerable, short-term toxicity, it remains imperative to address these concerns in future research as a prerequisite for successful clinical translation. One effective approach is the surface coating MSNs with biocompatible polymers, including polyethylene glycol (PEG), polyethyleneimine (PEI) and chitosan, which have been shown to prolong circulation time and minimize the cytotoxicity of MSNs 65-67. Alternatively, encapsulation of MSNs within liposomes can mask their reactive groups and prevent serum protein absorption, thereby enhancing bioavailability and biocompatibility while reducing immunogenicity 67-69. Furthermore, given the manufacturing demands of clinical application, comparable evaluation of various MSN synthesis methods should also be considered, in order to identify the optimal approach in terms of reproducibility, scalability, cost, and nanoparticle physiochemical properties.

In summary, we have generated rhCD137L-MSN, a CD137 agonist with an improved therapeutic potential that allows effective tumor control, while circumventing the hepatotoxicity often observed with agonistic anti-CD137 antibodies.

## Supplementary Material

Supplementary figures and table.

## Abbreviations

ALT: alanine aminotransferase
APC: antigen presenting cells
APTES: (3'-aminopropyl)trimethoxysilane
AST: aspartate aminotransferase
BFA: brefeldin A
BSA: bovine serum albumin
BS3: bis(sulfosuccinimidyl) suberate
CLL: chronic lymphocytic leukemia
CTAC: cetyltrimethylammonium chloride
DCs: dendritic cells
EBER: EBV-encoded RNA
EBV: Epstein-Barr virus
EGFR: epidermal growth factor receptor
FAP: fibroblast activation protein
FBS: fetal bovine serum
FDC: follicular dendritic cell
HCC: hepatocellular carcinoma
HCl: hydrochloric acid
HER2: human epidermal growth factor receptor 2
HL: Hodgkin lymphoma
HLA-DR: human leukocyte antigen - DR isotype
HRS: Hodgkin and Reed-Sternberg
HRP: horseradish peroxidase
HuIgG4: human IgG4
IFNγ: interferon γ
ICIs: immune checkpoint inhibitors
IL: interleukin
MSNs: mesoporous silica nanoparticles
NK: natural killer
NKTCL: NK/T cell lymphoma
NPC: nasopharyngeal carcinoma
rhCD137L: recombinant human CD137 ligand
RMS: rhabdomyosarcoma
PAGE: polyacrylamide gel electrophoresis
PBMC: peripheral blood mononuclear cells
PBS: phosphate buffered saline
PD-1: programmed cell death protein 1
PD-L1: programmed death-ligand 1
PEG: polyethylene glycol
PEI: polyethyleneimine
PLK1: polo-like kinase 1
PVDF: polyvinyldifluoride
sCD137: soluble CD137
SDS: sodium dodecylsulfate
ST: Strep-Tactin® XT 4Flow®
TAA: tumor-associated antigens
TBS: Tris-buffered saline
TEA: triethanolamine
TEOS: tetraethyl orthosilicate
TNF: tumor necrosis factor
TRAIL: TNF-related apoptosis-inducing ligand
Ure: urelumab
Uto: utomilumab
