# Supplementary material for: Development of a nanoparticle-based immunotherapy targeting CD137 for nasopharyngeal carcinoma treatment
Source: Theranostics. 2026 Jan 1;16(1):17–36. doi: 10.7150/thno.116136 (PMC12665109; doi:10.7150/thno.116136)
Supplement: Supplementary file 1 — Supplementary figures and table. [file thnov16p0017s1.pdf]

Supplementary Figures

Figure S1

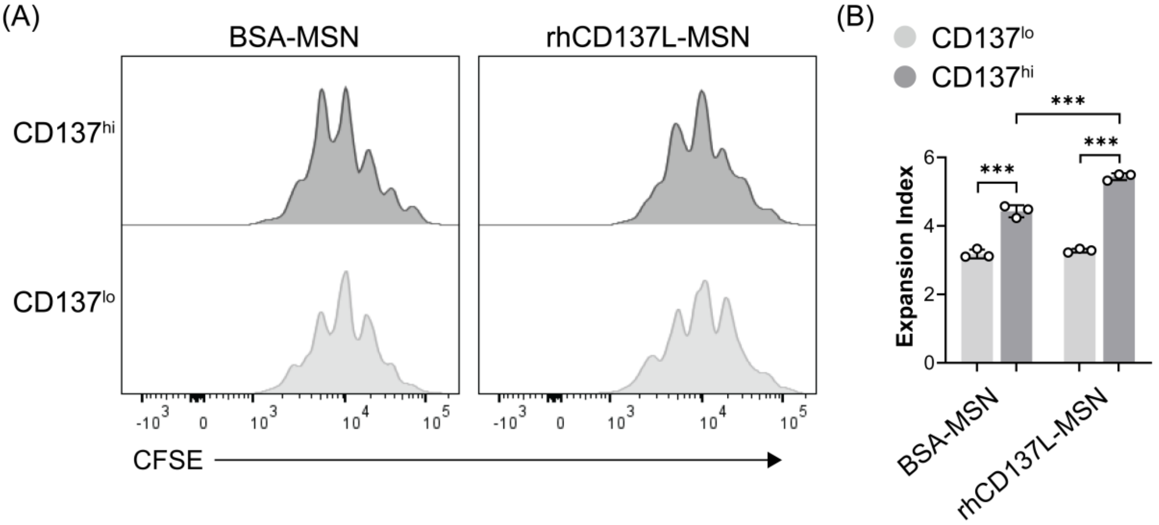

Figure S1. rhCD137L-MSNs enhance CD8$^+$ T cell proliferation. Human CD8$^+$ T cells were preactivated with human CD3/CD28 dynabeads for 24 h followed by stimulation with 5 µg/mL of rhCD137L-MSNs or BSA-MSNs. The cell proliferation was measured 3 days post treatment with the protein-MSN conjugates. The proliferation of CD137^{hi} and CD137^{lo} CD8$^+$ T cells in response to treatment with BSA-MSNs or CD137-MSNs are presented as (A) representative histograms and (B) are quantified by their expansion indices. Each symbol represents a biological replicate (n = 3). Data are shown as means ± SEM. Data are representative of two independent experiments with T cells of different donors. *** $p < 0.001$ using one-way ANOVA with Bonferroni's multiple comparison test. BSA: bovine serum albumin. MSN: mesoporous silica nanoparticle.

Figure S2

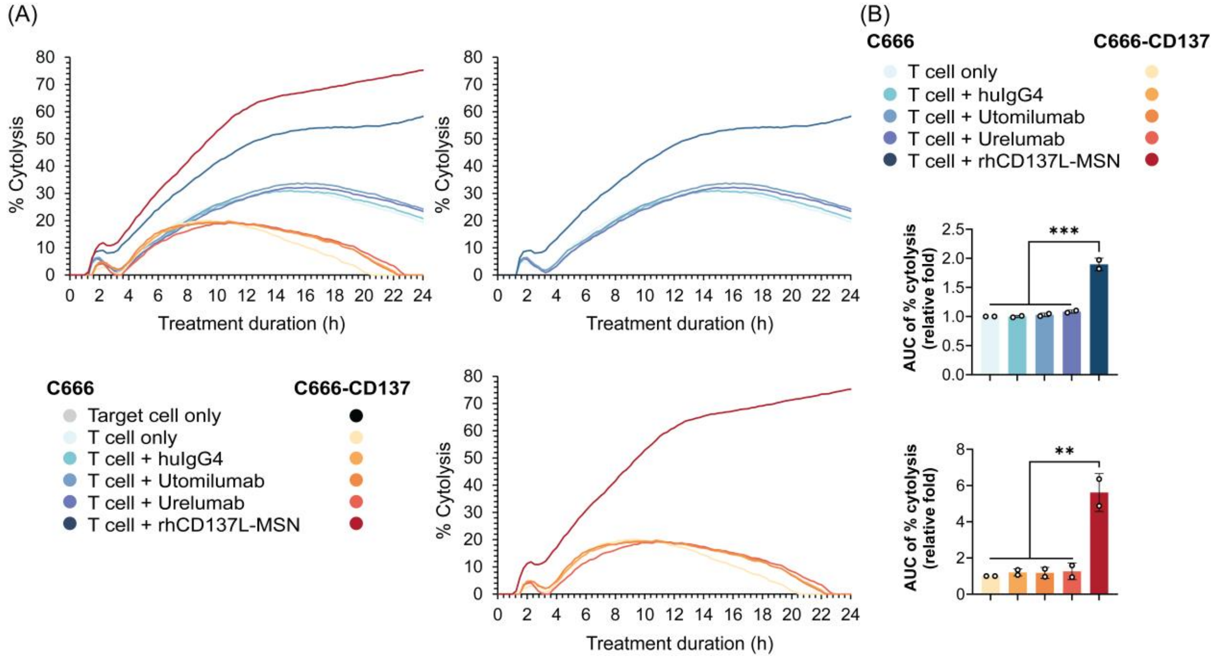

Figure S2. rhCD137L-MSNs show significantly greater efficacy in enhancing T cell cytotoxicity against CD137-expressing and CD137-null NPC cells than anti-CD137 antibodies. CD137-expressing (C666-CD137) and -null (C666) NPC cell lines were treated with suboptimally activated T cells and rhCD137L-MSNs or unconjugated anti-CD137 antibodies (urelumab or utomilumab) of equal molar concentration of receptor binding sites (197.5 nM) at 0 h. The tumor cytolysis results are illustrated in (A) as % cytolysis curves and in (B) as the area under the curve (AUC) of % cytolysis over 24 h post treatment. Each symbol represents a technical replicate. Data are shown as means ± SEM of duplicate wells. Data are representative of two independent experiments with T cells of different donors. ** $p < 0.01$, *** $p < 0.001$ using one-way ANOVA with Bonferroni's multiple comparison test. MSN: mesoporous silica nanoparticle.

Figure S3

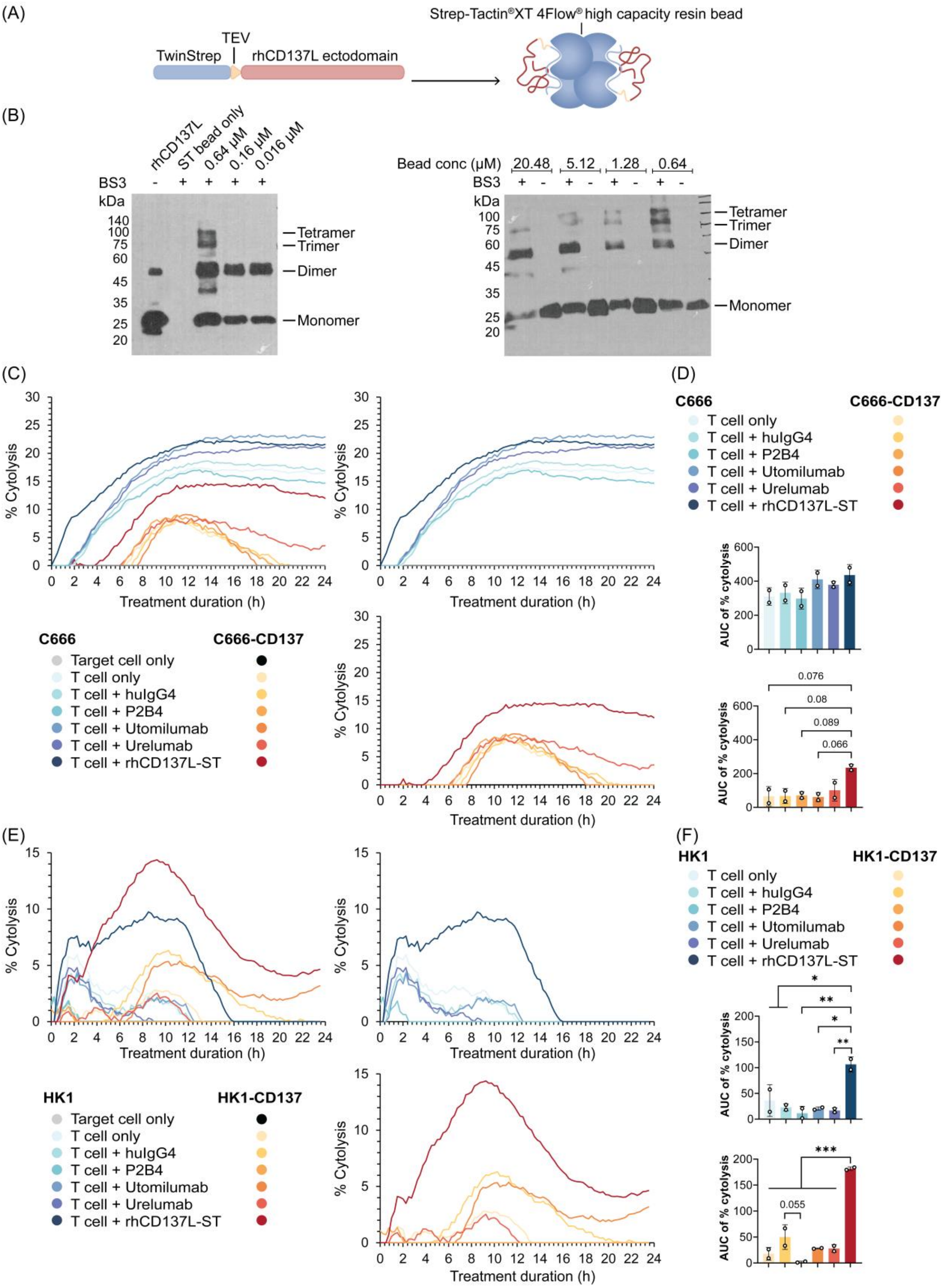

Figure S3. rhCD137L-ST induces a greater T cell cytotoxicity against CD137-expressing and CD137-null NPC cells than anti-CD137 antibodies. (A) Schematic representation of the protein domain of rhCD137L and its conjugation to StrepTactin®

XT 4Flow® resin beads. (B) Different amounts of the resin beads (Left: 0.016, 0.16 and 0.64 μM; Right: 0.64, 1.28, 5.12 and 20.48 μM) were used to conjugate 312.5 nM rhCD137L. The rhCD137L on the beads was crosslinked using bis(sulfosuccinimidyl)suberate linker (BS3) to preserve its oligomerization status prior to Western blot analysis using a mouse anti-human CD137L antibody (clone: 5F4). CD137-expressing and -null (C666 and HK1) NPC cell lines were treated with suboptimally activated T cells and rhCD137L-ST or unconjugated anti-CD137 antibodies (P2B4, urelumab or utomilumab) of equal molar concentration (70 nM) at 0 h. Two groups of NPC cell lines were used: (C-D) C666 and C666-CD137 and (E-F) HK1 and HK1-CD137. The tumor cytolysis results are illustrated in (C & E) as % cytolysis curves and in (D & F) as the area under the curve (AUC) of % cytolysis over 24 h post treatment. Each symbol represents a technical replicate. Data are shown as means ± SEM of duplicate wells. Data are representative of two independent experiments with T cells of different donors. Numbers above the bracket indicate p-values. * $p < 0.05$, ** $p < 0.01$, *** $p < 0.001$ using one-way ANOVA with Bonferroni's multiple comparison test. TEV: tobacco etch virus protease-cleavable linker.

Figure S4

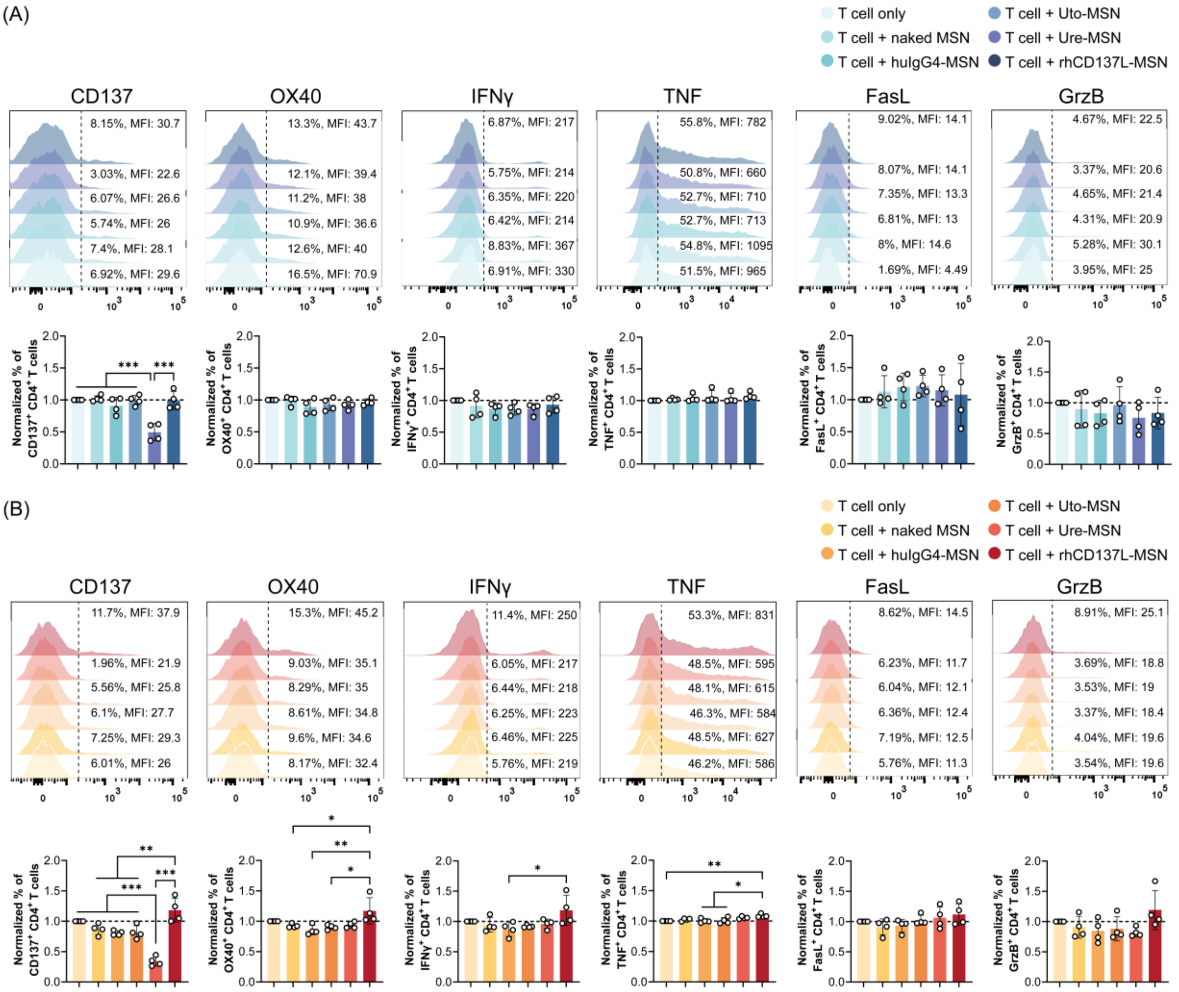

Figure S4. Treatment with rhCD137L-MSNs significantly enhances the activation and effector functions of CD4⁺ T cells that were cocultured with C666-CD137 cells. The expression levels of CD137, OX40, IFNγ, TNF, FasL and granzyme B (GrzB) in CD4⁺ T cells that were cocultured with (A) C666 and (B) C666-CD137 cells, were analyzed using flow cytometry. The expression levels of these markers in each treatment group were normalized to their levels in the T cell only treatment group. The results were obtained from T cells of four different donors, with each symbol representing an individual T cell donor ($n = 4$). A ratio of 1 (dashed line) indicates no difference between the T cell+treatment group and the T cell only control. Data are shown as means ± SEM. * $p < 0.05$, ** $p < 0.01$, *** $p < 0.001$ using one-way ANOVA with Bonferroni's multiple comparison test. Uto: utomilumab. Ure: urelumab. MSN: mesoporous silica nanoparticle.

Figure S5

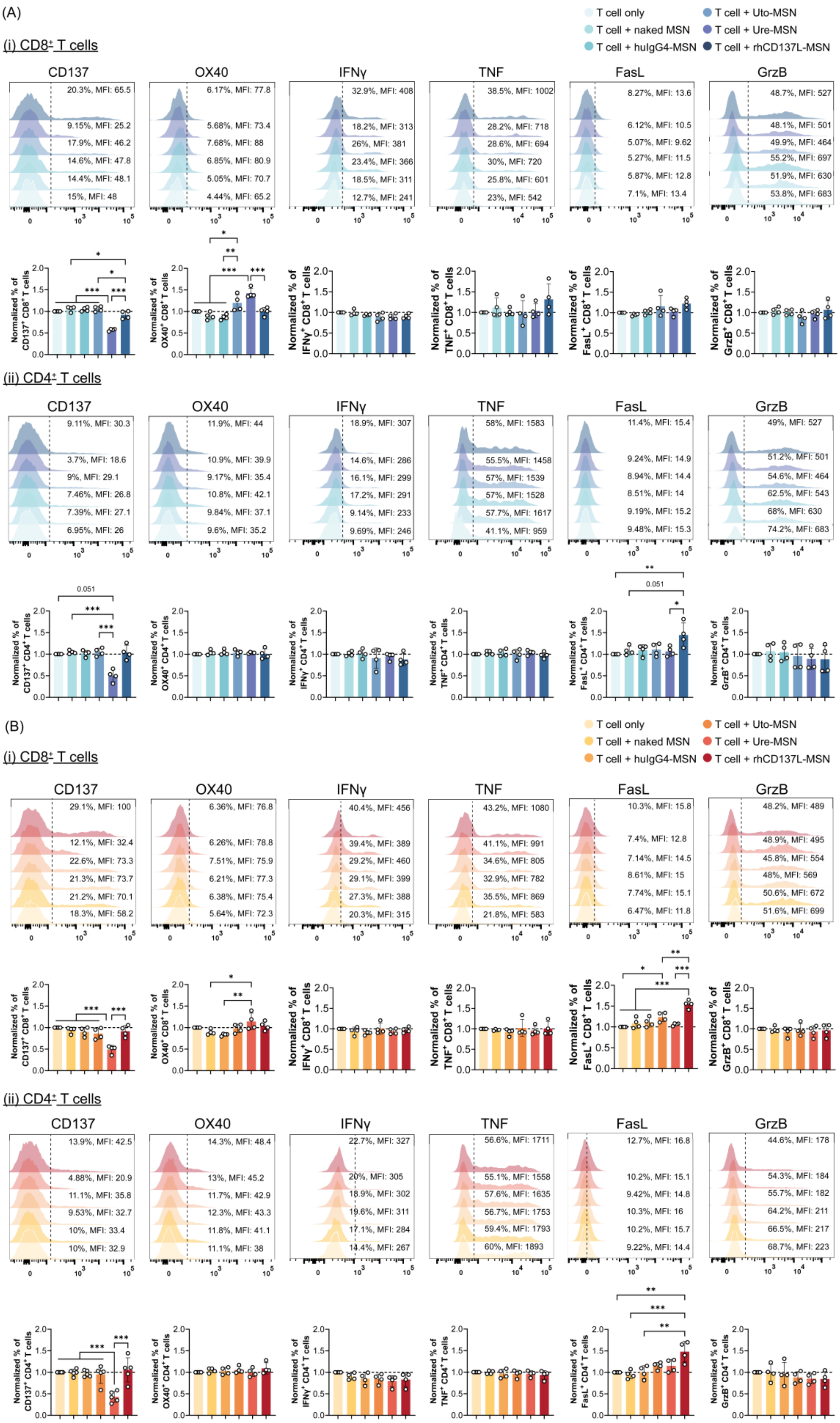
(A)
T cell only
T cell + naked MSN
T cell + huIgG4-MSN
T cell + Uto-MSN
T cell + Ure-MSN
T cell + rhCD137L-MSN
(i) CD8+ T cells
CD137
20.3%, MFI: 65.5
9.15%, MFI: 25.2
17.9%, MFI: 46.2
14.6%, MFI: 47.8
14.4%, MFI: 48.1
15%, MFI: 48
OX40
6.17%, MFI: 77.8
5.68%, MFI: 73.4
7.68%, MFI: 88
6.85%, MFI: 80.9
5.05%, MFI: 70.7
4.44%, MFI: 65.2
IFNγ
32.9%, MFI: 408
18.2%, MFI: 313
26%, MFI: 381
23.4%, MFI: 366
18.5%, MFI: 311
12.7%, MFI: 241
TNF
38.5%, MFI: 1002
28.2%, MFI: 718
28.6%, MFI: 694
30%, MFI: 720
25.8%, MFI: 601
23%, MFI: 542
FasL
8.27%, MFI: 13.6
6.12%, MFI: 10.5
5.07%, MFI: 9.62
5.27%, MFI: 11.5
5.87%, MFI: 12.8
7.1%, MFI: 13.4
GrzB
48.7%, MFI: 527
48.1%, MFI: 501
49.9%, MFI: 464
55.2%, MFI: 697
51.9%, MFI: 630
53.8%, MFI: 683
Normalized % of CD137+ CD8+ T cells
Normalized % of OX40+ CD8+ T cells
Normalized % of IFNγ+ CD8+ T cells
Normalized % of TNF+ CD8+ T cells
Normalized % of FasL+ CD8+ T cells
Normalized % of GrzB+ CD8+ T cells
(ii) CD4+ T cells
CD137
9.11%, MFI: 30.3
3.7%, MFI: 18.6
9%, MFI: 29.1
7.46%, MFI: 26.8
7.39%, MFI: 27.1
6.95%, MFI: 26
OX40
11.9%, MFI: 44
10.9%, MFI: 39.9
9.17%, MFI: 35.4
10.8%, MFI: 42.1
9.84%, MFI: 37.1
9.6%, MFI: 35.2
IFNγ
18.9%, MFI: 307
14.6%, MFI: 286
16.1%, MFI: 299
17.2%, MFI: 291
9.14%, MFI: 233
9.69%, MFI: 246
TNF
58%, MFI: 1583
55.5%, MFI: 1458
57%, MFI: 1539
57%, MFI: 1528
57.7%, MFI: 1617
41.1%, MFI: 959
FasL
11.4%, MFI: 15.4
9.24%, MFI: 14.9
8.94%, MFI: 14.4
8.51%, MFI: 14
9.19%, MFI: 15.2
9.48%, MFI: 15.3
GrzB
49%, MFI: 527
51.2%, MFI: 501
54.6%, MFI: 464
62.5%, MFI: 543
68%, MFI: 630
74.2%, MFI: 683
0.051
Normalized % of CD137+ CD4+ T cells
Normalized % of OX40+ CD4+ T cells
Normalized % of IFNγ+ CD4+ T cells
Normalized % of TNF+ CD4+ T cells
Normalized % of FasL+ CD4+ T cells
Normalized % of GrzB+ CD4+ T cells
(B)
T cell only
T cell + naked MSN
T cell + huIgG4-MSN
T cell + Uto-MSN
T cell + Ure-MSN
T cell + rhCD137L-MSN
(i) CD8+ T cells
CD137
29.1%, MFI: 100
12.1%, MFI: 32.4
22.6%, MFI: 73.3
21.3%, MFI: 73.7
21.2%, MFI: 70.1
18.3%, MFI: 58.2
OX40
6.36%, MFI: 76.8
6.26%, MFI: 78.8
7.51%, MFI: 75.9
6.21%, MFI: 77.3
6.38%, MFI: 75.4
5.64%, MFI: 72.3
IFNγ
40.4%, MFI: 456
39.4%, MFI: 389
29.2%, MFI: 460
29.1%, MFI: 399
27.3%, MFI: 388
20.3%, MFI: 315
TNF
43.2%, MFI: 1080
41.1%, MFI: 991
34.6%, MFI: 805
32.9%, MFI: 782
35.5%, MFI: 869
21.8%, MFI: 583
FasL
10.3%, MFI: 15.8
7.4%, MFI: 12.8
7.14%, MFI: 14.5
8.61%, MFI: 15
7.74%, MFI: 15.1
6.47%, MFI: 11.8
GrzB
48.2%, MFI: 489
48.9%, MFI: 495
45.8%, MFI: 554
48%, MFI: 569
50.6%, MFI: 672
51.6%, MFI: 699
Normalized % of CD137+ CD8+ T cells
Normalized % of OX40+ CD8+ T cells
Normalized % of IFNγ+ CD8+ T cells
Normalized % of TNF+ CD8+ T cells
Normalized % of FasL+ CD8+ T cells
Normalized % of GrzB+ CD8+ T cells
(ii) CD4+ T cells
CD137
13.9%, MFI: 42.5
4.88%, MFI: 20.9
11.1%, MFI: 35.8
9.53%, MFI: 32.7
10%, MFI: 33.4
10%, MFI: 32.9
OX40
14.3%, MFI: 48.4
13%, MFI: 45.2
11.7%, MFI: 42.9
12.3%, MFI: 43.3
11.8%, MFI: 41.1
11.1%, MFI: 38
IFNγ
22.7%, MFI: 327
20%, MFI: 305
18.9%, MFI: 302
19.6%, MFI: 311
17.1%, MFI: 284
14.4%, MFI: 267
TNF
56.6%, MFI: 1711
55.1%, MFI: 1558
57.6%, MFI: 1635
56.7%, MFI: 1753
59.4%, MFI: 1793
60%, MFI: 1893
FasL
12.7%, MFI: 16.8
10.2%, MFI: 15.1
9.42%, MFI: 14.8
10.3%, MFI: 16
10.2%, MFI: 15.7
9.22%, MFI: 14.4
GrzB
44.6%, MFI: 178
54.3%, MFI: 184
55.7%, MFI: 182
64.2%, MFI: 211
66.5%, MFI: 217
68.7%, MFI: 223
Normalized % of CD137+ CD4+ T cells
Normalized % of OX40+ CD4+ T cells
Normalized % of IFNγ+ CD4+ T cells
Normalized % of TNF+ CD4+ T cells
Normalized % of FasL+ CD4+ T cells
Normalized % of GrzB+ CD4+ T cells

Figure S5. Activation and functional profiles of T cells that were cocultured with HK1 and HK1-CD137 cells. The expression levels of CD137, OX40, IFNγ, TNF, FasL and granzyme B (GrzB) in $CD8^+$ and $CD4^+$ T cells that were cocultured with (A) HK1 and (B) HK1-CD137 cells, were analyzed using flow cytometry. The expression levels of these markers in each treatment group were normalized to their levels in the T cell only treatment group. The results were obtained from T cells of four different donors, with each symbol representing an individual T cell donor (n = 4). A ratio of 1 (dashed line) indicates no difference between T cell+treatment group and T cell only control. Data are shown as means ± SEM. * $p < 0.05$, ** $p < 0.01$, *** $p < 0.001$ using one-way ANOVA with Bonferroni's multiple comparison test. Uto: utomilumab. Ure: urelumab. MSN: mesoporous silica nanoparticle.

Figure S6

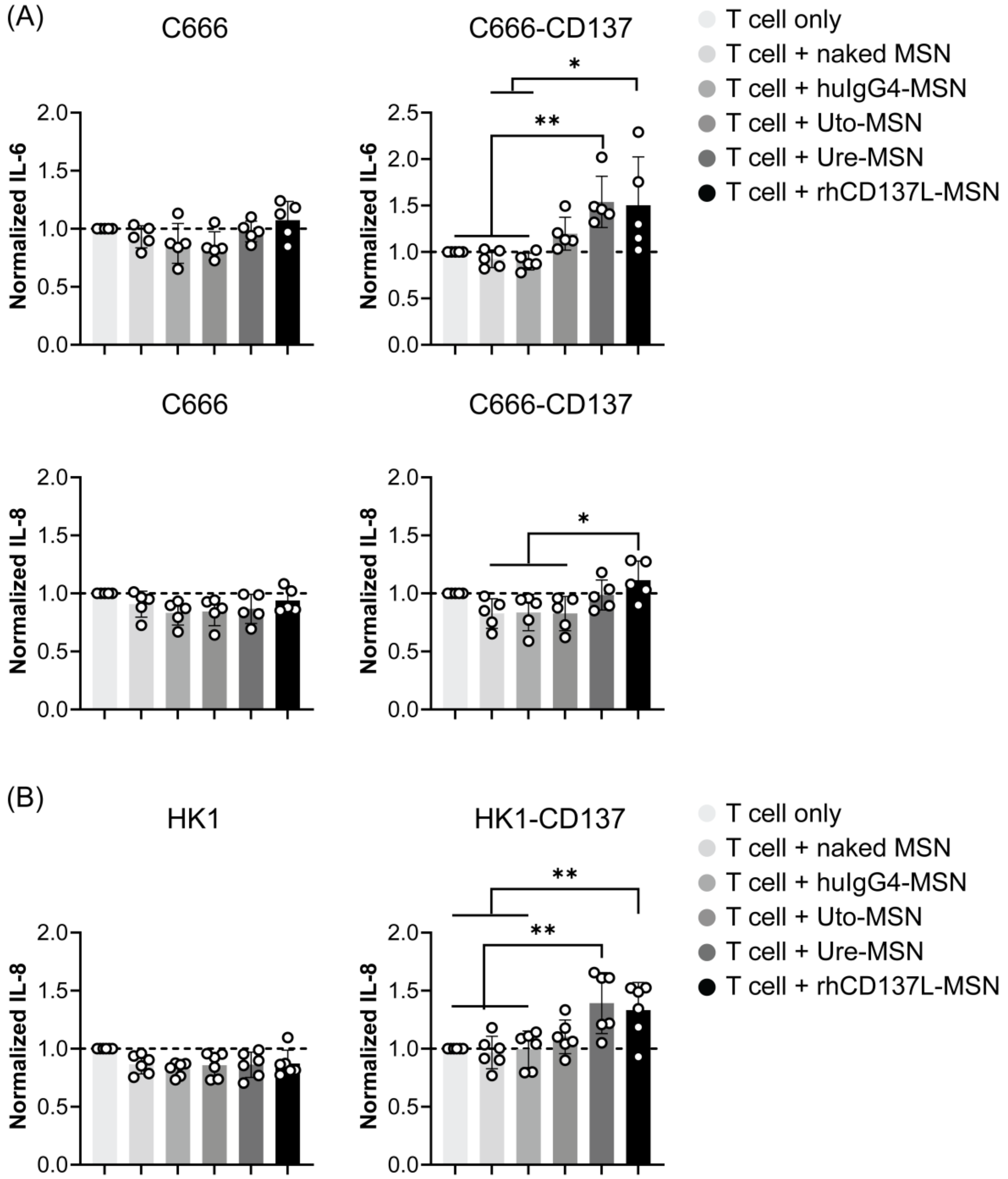

Figure S6. Treatment with rhCD137L-MSNs enhances the production of IL-6 and/or IL-8 by C666-CD137 and HK1-CD137 cells. CD137-expressing (C666-CD137 or HK1-CD137) NPC cells and their control cells (C666 or HK1) were tested for their production of IL-6 and/or IL-8 under different treatment conditions using ELISA. The levels of (A) IL-6 and IL-8 secreted by C666 and C666-CD137 cells, and (B) IL-8 by HK1 and HK1-CD137 cells, were normalized to the levels of respective tumor cells treated with T cells alone. Each symbol represents a different T cell donor (n = 5-6). Data are shown as means ± SEM. Data are representative of two independent experiments. A ratio of 1 (dashed line) indicates no difference between T cell+treatment group and T cell only control. * $p < 0.05$, ** $p < 0.01$ using one-way

ANOVA with Bonferroni's multiple comparison test. Uto: utomilumab. Ure: urelumab. MSN: mesoporous silica nanoparticle.

Figure S7

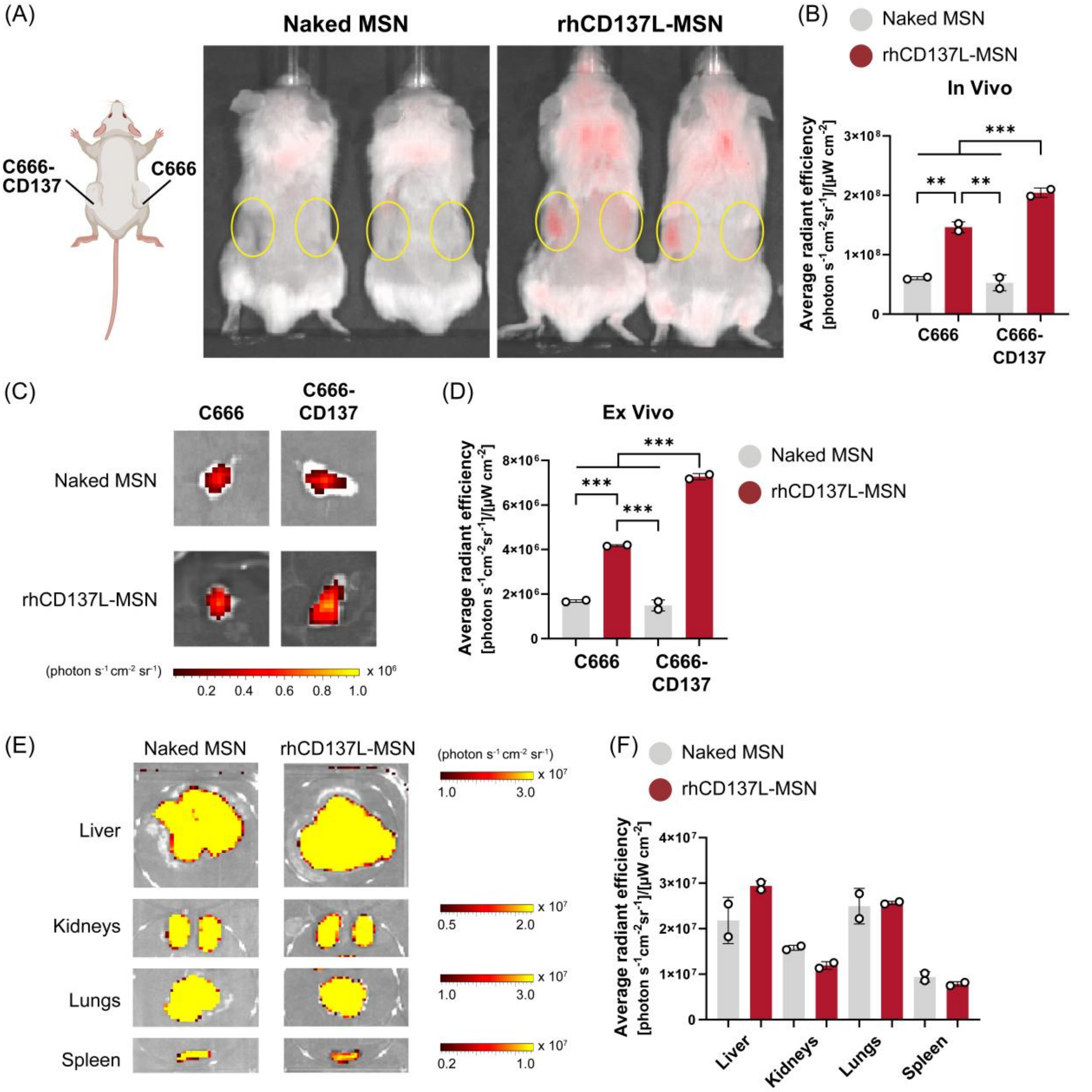

Figure S7. Surface-functionalization of MSNs with rhCD137L promotes their accumulation in CD137-expressing tumors. PBMC-humanized NOD-scid (JInx) bearing C666 and C666-CD137 tumors on their right and left flanks, respectively, were administrated with a single intravenous dose (10 mg/kg) of Cy5.5-labelled rhCD137L-MSNs or naked MSNs. Bioluminescent imaging of the whole mice, isolated tumors and main organs were performed 48 h post-injection. (A) Representative images showing the localization of rhCD137L-MSNs or naked MSNs at the tumor sites (yellow circle). (B) Quantification of average radiant efficiency of *in vivo* tumors. (C) Representative fluorescence images and (D) average radiant efficiency of excised tumors. (E) Representative fluorescence images and (F) average radiant efficiency of livers, kidneys, lungs and spleens. Each symbol represents one mouse (n = 2). All data are shown as means ± SEM. Data are representative of two independent experiments. ** $p < 0.01$, *** $p < 0.001$ using one-way ANOVA with Bonferroni's multiple comparison test. MSN: mesoporous silica nanoparticle.

Figure S8

(A)

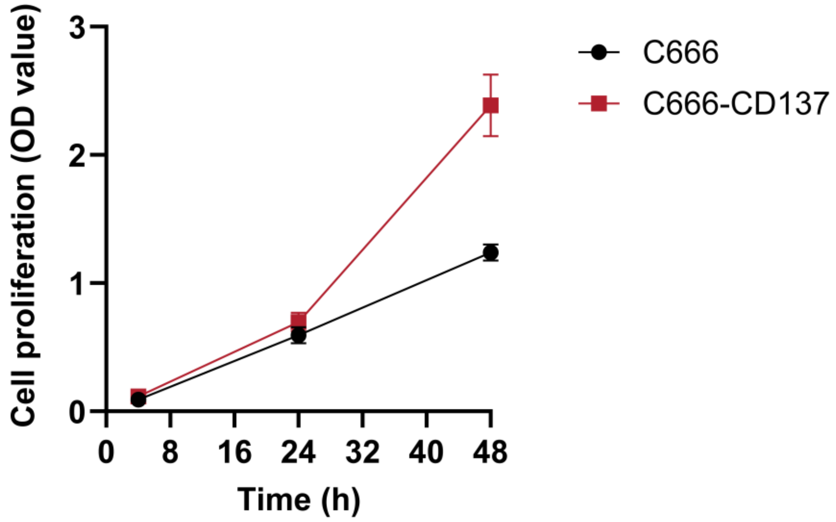

(B)

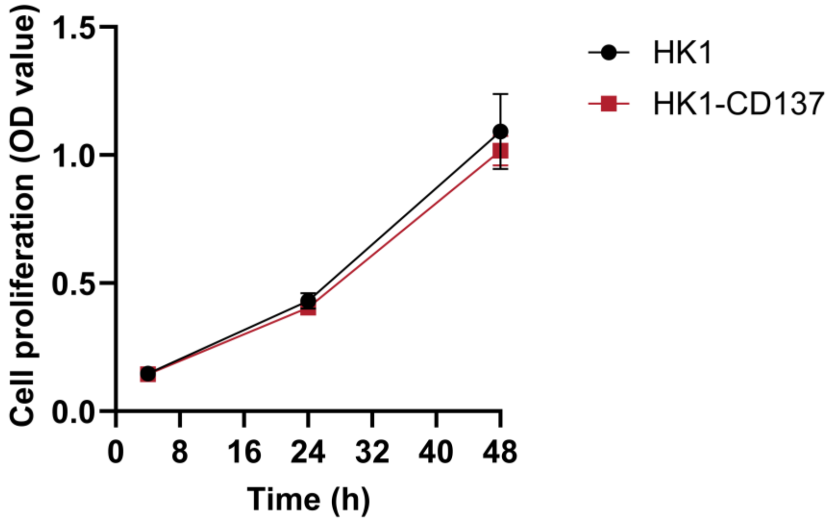

Figure S8. Proliferation rates of CD137-expressing and -null NPC cell lines. The proliferation of CD137-expressing and CD137-null NPC cell lines was measured at 4, 24 and 48 h using a CCK8 assay. Results for (A) C666 and C666-CD137 cells, and (B) HK1 and HK1-CD137 cells are shown. Data are representative of two independent experiments. OD: Optical density.

Figure S9

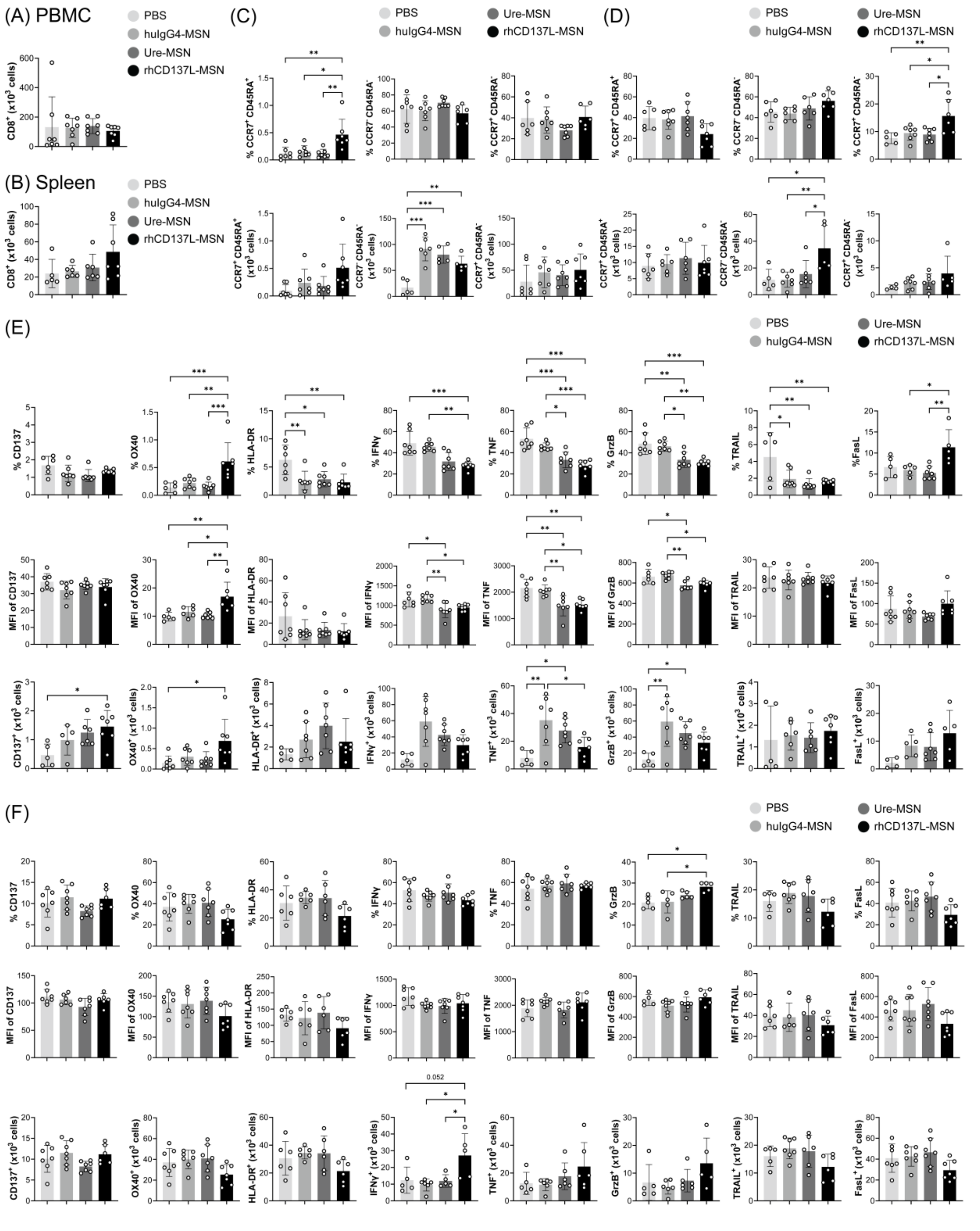

Figure S9. Phenotypic analysis of peripheral and splenic CD8$^+$ T cells in C666/C666-CD137 tumor-bearing humanized mice. Number of CD8$^+$ T cells in (A) peripheral blood and (B) spleen of humanized mice bearing C666/C666-CD137 tumors. CD8$^+$ T cells in the state of naïve (CCR7$^+$ CD45RA$^+$), effector memory (CCR7$^-$ CD45RA$^-$), and central memory (CCR7$^+$ CD45RA$^-$) were examined in (C) peripheral blood and (D) spleen. Data are shown in % population and numbers of cells. Expression levels of CD137, OX40, HLA-DR, IFNγ, TNF, granzyme B (GrzB), TRAIL

and FasL of tumor-infiltrating $CD8^+$ T cells in (E) peripheral blood and (F) spleen were analyzed in terms of % population, mean fluorescence intensity (MFI), and absolute numbers of cells. Each symbol represents one mouse (n = 5-6). All data are shown as means ± SEM. Data are representative of two independent experiments. * $p < 0.05$, ** $p < 0.01$, *** $p < 0.001$ using one-way ANOVA with Bonferroni's multiple comparison test. PBS: phosphate buffered saline. Ure: urelumab. MSN: mesoporous silica nanoparticle.

Figure S10

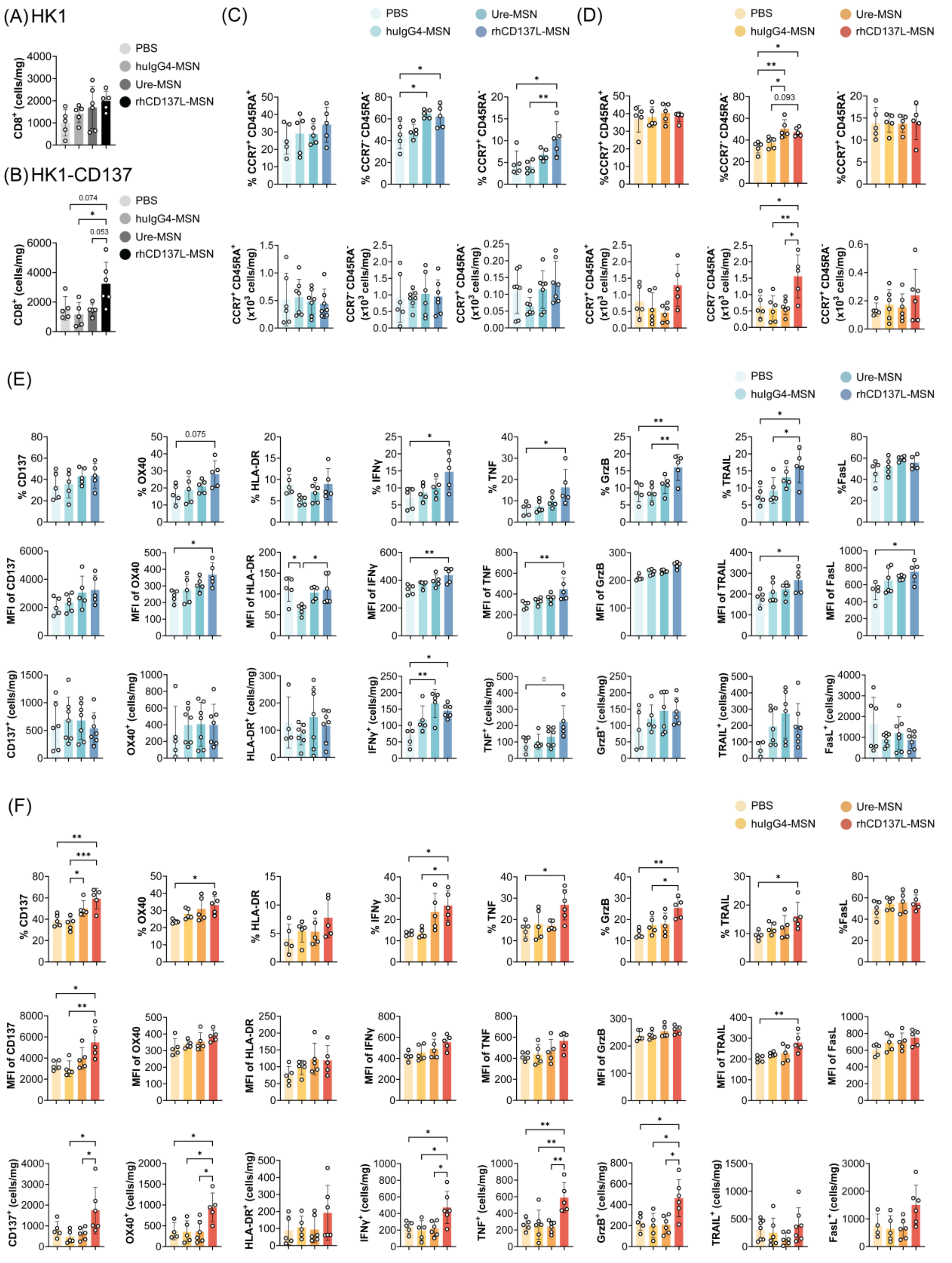

Figure S10. Increased tumor infiltration of activated and polyfunctional CD8$^+$ T cells with rhCD137L-MSN treatment. Number of CD8$^+$ T cells in (A) HK1 and (B) HK1-CD137 tumors of the different treatment groups. CD8$^+$ T cells in the state of naïve (CCR7$^+$ CD45RA$^+$), effector memory (CCR7$^-$ CD45RA$^-$), and central memory (CCR7$^+$

CD45RA$^-$) were examined in (C) HK1 and (D) HK1-CD137 tumors. The data are shown in % population and numbers of cells. Expression levels of CD137, OX40, HLA-DR, IFNγ, TNF, granzyme B (GrzB), TRAIL and FasL of tumor-infiltrating CD8$^+$ T cells in (E) HK1 and (F) HK1-CD137 tumors were analyzed in terms of % population, mean fluorescence intensity (MFI), and absolute numbers of cells. Each symbol represents one mouse (n = 5-6). All data are shown as means ± SEM. Data are representative of two independent experiments * $p < 0.05$, ** $p < 0.01$, *** $p < 0.001$ using one-way ANOVA with Bonferroni's multiple comparison test. PBS: phosphate buffered saline. Ure: urelumab. MSN: mesoporous silica nanoparticle.

Supplementary Table

		NPC
**Total**		113
**Sex**		
	M	77 (68.1%)
	F	36 (31.9%)
**Age**		48 (10-75)

Table S1. The baseline characteristics of NPC patients.
